# Targeting lymphoid-derived IL-17 signaling to delay skin aging

**DOI:** 10.1038/s43587-023-00431-z

**Published:** 2023-06-08

**Authors:** Paloma Solá, Elisabetta Mereu, Júlia Bonjoch, Marta Casado-Peláez, Neus Prats, Mònica Aguilera, Oscar Reina, Enrique Blanco, Manel Esteller, Luciano Di Croce, Holger Heyn, Guiomar Solanas, Salvador Aznar Benitah

**Affiliations:** 1 Institute for Research in Biomedicine, Barcelona Institute of Science and Technology, Barcelona, Spain; 2 Josep Carreras Leukemia Research Institute, Badalona, Spain; 3 Centre for Genomic Regulation, Barcelona Institute of Science and Technology, Barcelona, Spain; 4 ICREA, Catalan Institution for Research and Advanced Studies, Barcelona, Spain

**Keywords:** Stem cells, Ageing

## Abstract

Skin aging is characterized by structural and functional changes that contribute to age-associated frailty. This probably depends on synergy between alterations in the local niche and stem cell-intrinsic changes, underscored by proinflammatory microenvironments that drive pleotropic changes. The nature of these age-associated inflammatory cues, or how they affect tissue aging, is unknown. Based on single-cell RNA sequencing of the dermal compartment of mouse skin, we show a skew towards an IL-17-expressing phenotype of T helper cells, γδ T cells and innate lymphoid cells in aged skin. Importantly, in vivo blockade of IL-17 signaling during aging reduces the proinflammatory state of the skin, delaying the appearance of age-related traits. Mechanistically, aberrant IL-17 signals through NF-κB in epidermal cells to impair homeostatic functions while promoting an inflammatory state. Our results indicate that aged skin shows signs of chronic inflammation and that increased IL-17 signaling could be targeted to prevent age-associated skin ailments.

## Abstract (web summary)

Solá, Mereu and colleagues describe a chronic inflammatory state in the aging mouse skin that is characterized by IL-17 production by dermal lymphoid cells and demonstrate the potential of inhibition of IL-17 signaling in protection against skin aging.

## Main

Aging is caused by cellular damage accumulation due to ineffective metabolism, circadian clock rewiring and increased systemic inflammation, leading to dysfunction and eventual organismal collapse when challenged^1–6^.

The skin contains a multilayered epidermis interspersed with hair follicles and sebaceous glands. The outermost layer of the skin, the epidermis, is a stratified epithelium that forms an impermeable protection for the organism. Daily renewal of the epidermis is fueled by interfollicular epidermal stem cells whereas hair follicle stem cells maintain hair follicles by periodical generation of new hair shafts^7^. In the dermis, an organized mesh of extracellular matrix (ECM) embedded with distinct cell types forms the niche for all epidermal stem cells^7^. Epidermal aging is characterized by alterations in its regenerative potential and barrier functions^8–12^. Moreover, age-related changes in the dermal cellular composition and ECM properties affect its functionality, both as a structural scaffold and as a niche for epidermal stem cells^13–18^. Collectively these changes lead to a slower epidermal turnover, breaching of the barrier and lower quality of wound healing, all of which contribute to increasing the incidence of infections and chronic traits in the elderly^19–21^.

Previous single-cell RNA sequencing (scRNA-seq) studies focused on defining changes in skin cell types during homeostasis, aging and disease suggest that some immune cell types change their abundance or behavior during aging in skin^13,22–29^. However, much remains unknown regarding the relationship between immune cells of the dermis and other dermal and epidermal cells during aging.

Here we unbiasedly profiled and characterized the single-cell transcriptome of dermal cells in aged skin in mice. CD4^+^ T helper (T_H_) cells, γδ T cells and innate lymphoid cells (ILCs) showed the most prominent changes during aging and appeared to orchestrate many of the alterations observed in the aged skin. Specifically, these cells became polarized towards an IL-17-producing phenotype, strongly contributing to the inflammatory environment found in aged skin. Importantly, in vivo blockade of IL-17 signaling during the aging process delayed the development of several hallmarks of skin aging, resembling the state of adult skin.

## Results

### Aged dermal cells reveal cell type- and age-specific changes

**Fig. 1:**
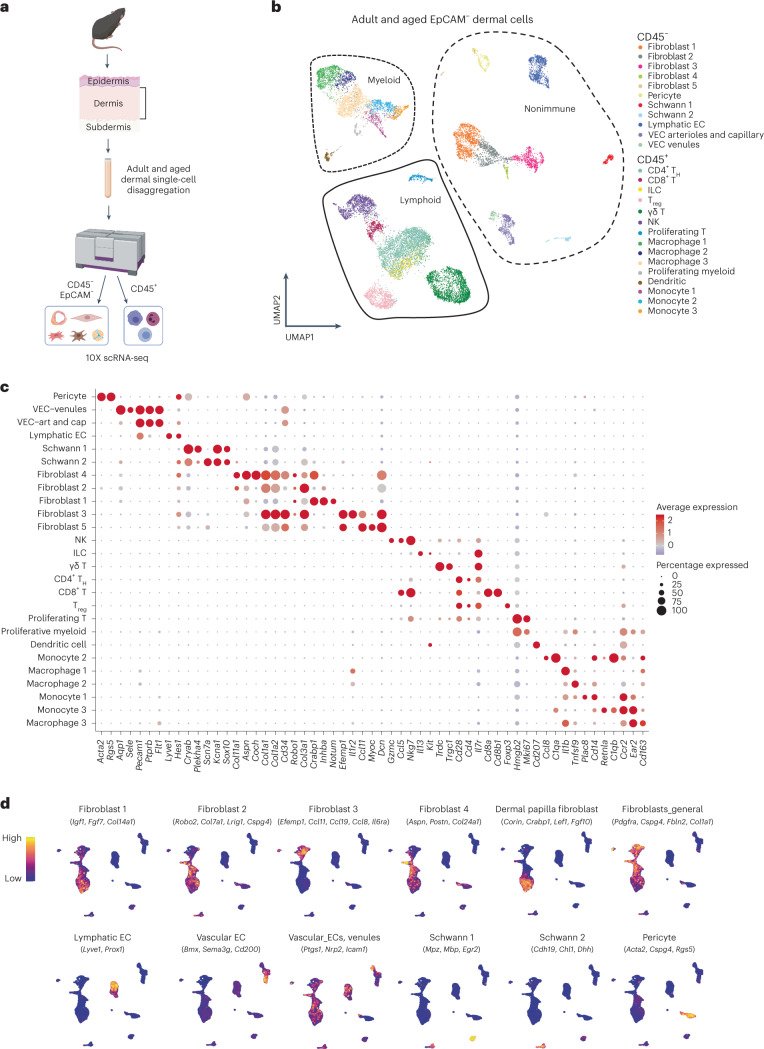
Dermal cell characterization by 10X scRNA-seq. **a**, Workflow used to obtain dermal cells of adult and aged mouse back skin. Single-cell suspensions were enriched separately for EpCAM^–^CD45^–^ and CD45^+^ cells by FACS. Transcriptomes of sorted single cells were then analyzed by 10X scRNA-seq. For CD45^+^ cells, *n* = 7 mice for the adult group and *n* = 4 mice for the aged group, with three technical replicates; for CD45^–^EpCAM^–^ cells, *n* = 2 mice for the control group and *n* = 2 mice for the aged group, with two technical replicates. Created with BioRender.com. **b**, UMAP visualization of all adult and aged dermal cells analyzed by 10X scRNA-seq. **c**, Dot plot showing discriminatory markers for each cell type, subtype and state found in **b**. **d**, UMAP visualization of nonimmune cell subtype-specific signatures. EC, endothelial cells; VEC, vascular endothelial cells.

**Extended Data Fig. 1:**
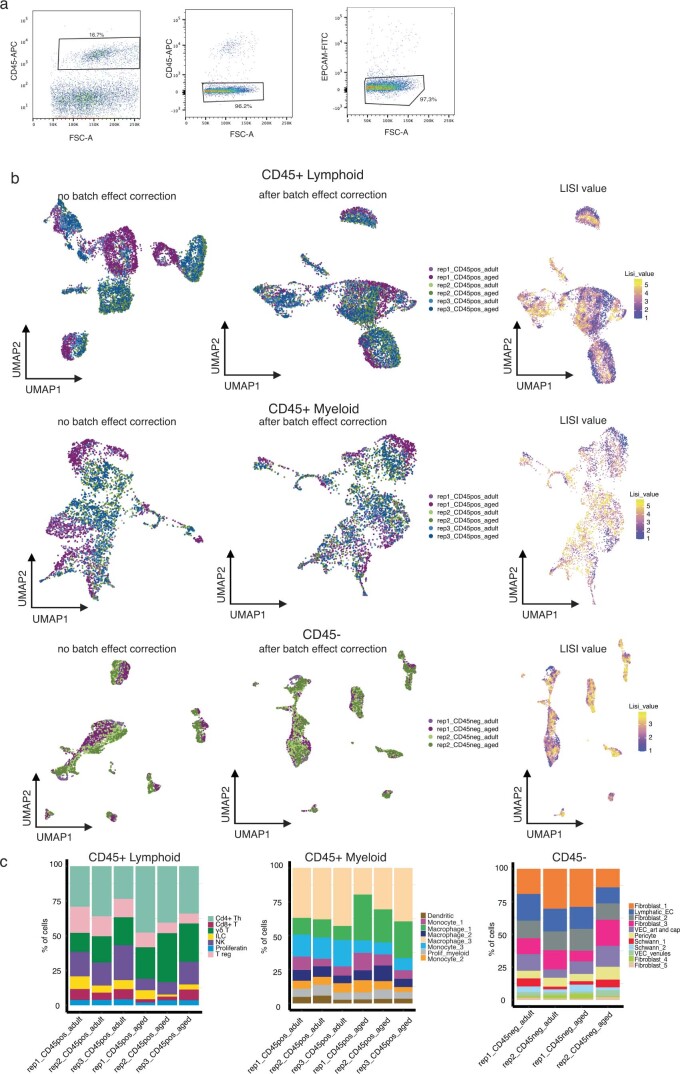
Sorting strategy and quality controls of scRNA-seq of adult and aged dermis. **a**, Flow cytometry plots representative of the sorting strategy followed to obtain CD45+ and CD45–/EpCAM– cells for 10× scRNA-seq. **b**, UMAP representation of CD45+ Lymphoid (upper panel), CD45+ Myeloid (middle panel) and CD45- (lower panel) cells showing replicate contribution, batch effect correction and good sample mixing calculated with LISI(Korsunsky, Millard et al. 2019). **c**, Bar plots showing percentage of total cells divided into each cell type per replicate, CD45+ Lymphoid (left panel), CD45+ Myeloid (middle panel) and CD45- (right panel) cells.

We analyzed the nonepithelial (negative for epithelial cell adhesion molecule (EpCAM^–^)) dermal population of dorsal skin from aged (80- to 90-week-old) and adult (17- to 25-week-old) mice by scRNA-seq. We removed epidermal cells enzymatically and isolated dermal cells by fluorescence-activated cell sorting (FACS) (Fig. 1a and Extended Data Fig. 1a). To maximize sampling of less abundant immune cells, we enriched by FACS for CD45^+^ cells and sequenced these separately from other dermal cells (EpCAM^–^CD45^–^) (Extended Data Fig. 1a). Using the 10X Genomics platform (v.3) we characterized 11,940 cells for CD45^+^ cells and 5,213 for their CD45^–^EpCAM^–^ counterparts. After batch correction and data integration, we verified good sample mixing^30^ and representation of all replicates across all populations (Extended Data Fig. 1b,c). We clustered all cells by generation of a shared nearest-neighbor graph using the Louvain algorithm^31^ and visualized clustering with uniform manifold approximation and projection (UMAP). We then performed differential gene expression between cell populations to obtain cluster markers (Fig. 1b and Supplementary Table 1) and plotted discriminatory population markers to ensure correct clustering (Fig. 1c). In UMAP visualization we observed three major groups of clusters pertaining to nonimmune (CD45^–^EpCAM^–^), immune myeloid and immune lymphoid lineages (both CD45^+^) (Fig. 1b,c).

#### Nonimmune cells

Although we detected clusters for endothelial cells, along with pericytes and Schwann cells (Fig. 1b–d), fibroblasts were the most abundant CD45^–^EpCAM^–^ cell type and were separated into five clusters containing subtypes with distinct features (Fig. 1d). Some of these clusters showed high similarity to well-defined fibroblast subtypes regarding marker expression—cluster 1 was reticular like, cluster 2 papillary like and cluster 3 proinflammatory^23,24,32–34^. Cluster fibroblast 1 included markers of dermal papilla cells^35^, a specialized cluster of fibroblasts located immediately beneath hair follicles that is essential for modulation of the hair follicle cycle (Fig. 1c,d).

**Extended Data Fig. 2:**
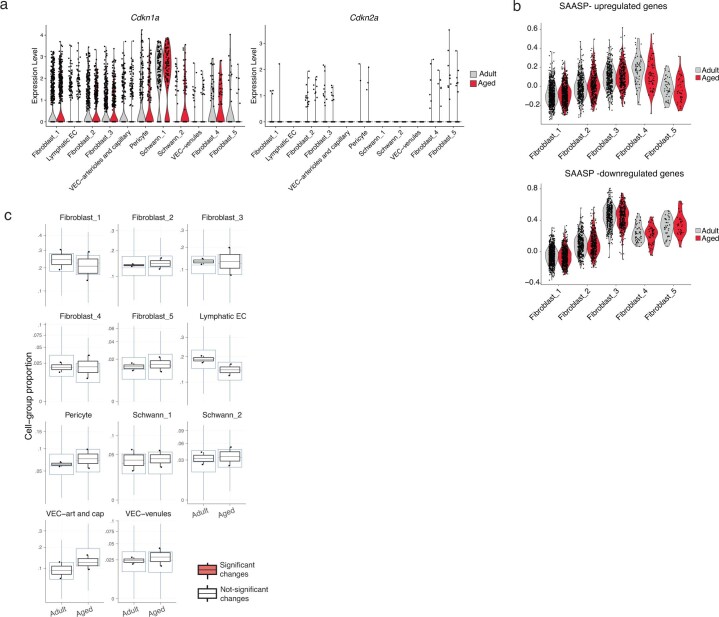
Non-immune dermal cell populations during aging. **a**, Violin plots showing expression of senescent-associated markers *Cdkn1a* (p21) and *Cdkn2a* (p16) in non-immune (CD45-/EpCAM-) cell populations. **b**, Violin plots showing average expression of the skin aging-associated secreted protein (SAASP) signature comparing adult and aged fibroblast populations(Waldera Lupa, Kalfalah et al. 2015). **c**, Box plots showing changes in proportion of non-immune (CD45-) cell populations in adult and aged conditions obtained with *sccomp*(Mangiola 2022) obtained with the scRNA-seq data. Error bars indicate the 95% credible intervals, center line is the median. The black boxplots represent the observed data, while blue ones indicate the posterior predictive check of the model. Boxes colored in orange indicate statistically significant differences in cell proportion between conditions (FDR < 0.025 using Benjamini-Hochberg procedure to control for multiple testing).

With fibroblasts being the most predominant cell type in the dermis, their age-associated alterations probably affect neighboring cells. To further understand their age-related changes we analyzed the expression of senescence-related markers. The accumulation of senescent cells is suggested to be a key mechanism underlying aging^3^. These nonproliferating senescent cells generate a proinflammatory environment by secretion of an array of cytokines and ECM remodeling enzymes among others. Although detection of senescent cells in vivo is challenging, transcriptional upregulation of *Cdkn2a* (which encodes p16) and/or *Cdkn1a* (p21) is considered a sign of senescence^3^. We did not observe increased expression of these genes in our dermal nonimmune clusters, which include fibroblasts (Extended Data Fig. 2a and Supplementary Table 2). Besides, there was no upregulation of *Ifna* and *Ifnb1*, additional bona fide markers of senescence^36^. Moreover, there was no global difference in expression of the skin aging-associated secreted protein signature^37^ in any of the fibroblast populations with aging (Extended Data Fig. 2b). This suggests that there is no increased expression of senescence in our dataset, which could have been caused by either a lack of sufficient depth to detect these marker changes in our experimental setup or to senescence not being a major driver of fibroblast aging in the age group analyzed.

The nonimmune compartment showed stable cell proportions during aging (Extended Data Fig. 2c). This was accompanied by transcriptomic changes in some populations, including fibroblasts 1 and 3 (Supplementary Table 2), which pointed towards an increase in their global proinflammatory state. Nevertheless, the changes observed by 10X scRNA-seq in these populations were subtle and did not allow for in-depth analysis of their specific age-related alterations.

#### Immune cells

**Fig. 2:**
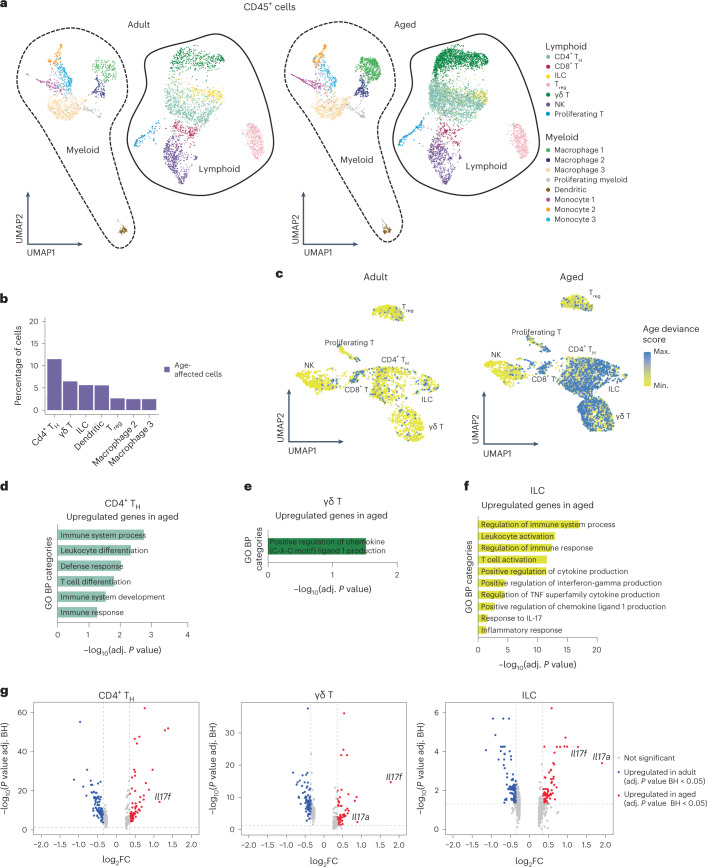
Lymphoid immune cell subsets show increased IL-17A/F-related signaling. **a**, UMAP representation of immune (CD45^+^) cells. **b**, Bar plot with the percentage of cells affected by aging in each immune dermal cell type (only cell types with higher percentage are shown here, for visual clarity). **c**, UMAP showing age-deviance score in adult (left) and aged (right) dermal lymphoid cells. **d**–**f**, Plots of selected GO categories in biological processes (BP) analysis for genes upregulated during aging in CD4^+^ T_H_ cells (**d**), γδ T cells (**e**) and ILCs (**f**). The *x* axis represents –log_10_ of the adjusted *P* value for each depicted GO category. **g**, Volcano plots showing differentially expressed genes between aged and adult CD4^+^ T_H_ cells, γδ T cells and ILCs. *Il17a* and *Il17f* are highlighted whenever found to be differentially expressed in a statistically significant manner between ages. BH, Benjamini–Hochberg; FC, fold change.

**Extended Data Fig. 3:**
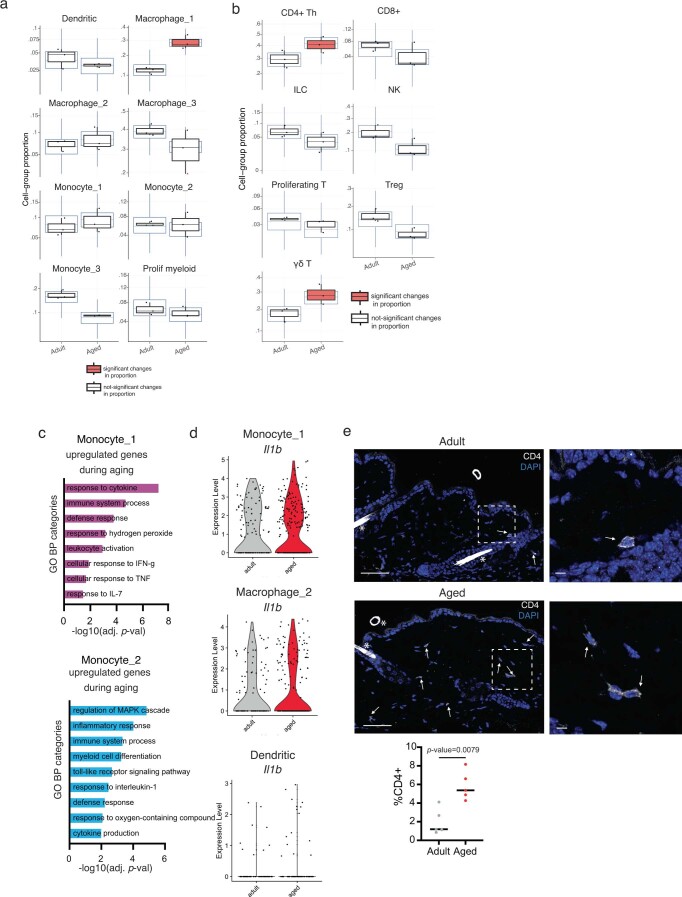
Changes observed in adult vs. aged dermis. **a,b**, Box plots showing changes in proportion of (**a**) myeloid and (**b**) lymphoid cells in adult and aged conditions obtained with *sccomp*(Mangiola 2022) obtained with the scRNA-seq data. Error bars indicate the 95% credible intervals, center line is the median. The black boxplots represent the observed data, while blue ones indicate the posterior predictive check of the model. Boxes colored in orange indicate statistically significant differences in cell proportion between conditions (FDR < 0.025 using Benjamini-Hochberg procedure to control for multiple testing) and red triangles outside the boxes mark outliers. **c**, Plots of selected GO categories belonging to Biological Processes (BP) analysis for genes upregulated in monocyte_1 cluster (upper panel) and monocyte_2 cluster (lower panel) upon aging. The x axis represents –(log_10_) of the adjusted *P*-value for each depicted GO category. **d**, Violin plots showing expression levels of *Il1b* in cells belonging to clusters monocyte_1, macrophage_2, and dendritic cells in adult and aged conditions. **e**, Immunostaining for CD4 in adult (upper panel) and aged (lower panel) back skin. White arrows point to positive cells. Bar = 50 μm. Insets are shown in the right panel next to each condition, bar = 10 μm. * = unspecific hair shaft autofluorescence. N = 5 individual mice. Quantification shows percentage of positive cells. Brightness and contrast were adjusted only for visualization purposes. Quantifications were done in raw images.

Visual representation of immune cell clusters rendered two major cell groups, myeloid and lymphoid (Fig. 2a). We observed statistically significant changes in proportions in some immune populations during aging (Extended Data Fig. 3a,b).

To measure the effect of aging on the transcriptome of immune cells in a global and unbiased manner, we developed a deep learning model that quantifies the deviance of each immune cell type in aging. We assumed that the most age-affected cells would present the most altered transcriptomic changes compared with their adult counterparts and, therefore, would be more distant in multidimensional gene expression space. We used our 10X scRNA-seq data from adult mice as a reference to train our model for each different cell type signature and then evaluated the similarity between adult and aged cells within each cell type. Cells with the highest deviance in their identity were those accumulating more differences between ages. Notably, we found that CD4^+^ T_H_ cells, γδ T cells, ILCs and dendritic cells were the types with the highest age deviance, showing the highest proportion of cells affected by aging (Fig. 2b). This suggests that lymphoid cell subsets present the most predominant age-related changes. Focusing on lymphoid cells, UMAP representation of exclusively lymphoid cells showed that CD4^+^ T_H_ cells, γδ T cells and ILCs possess a large proportion of cells with a high age-deviance score (Fig. 2c).

Therefore, because cell proportion changes accompany transcriptomic changes in some dermal immune cells during aging, we focused our further analysis on these immune cell types.

### Aged dermal myeloid cells upregulate proinflammatory genes

Dermal myeloid cells balance pro- and anti-inflammatory functions in skin in a context-dependent manner^38^. Specifically, they scan the tissue to detect antigens, orchestrate an early response to pathogens and trigger the first steps in the wound-healing response^39^. We detected eight clusters of myeloid cells: macrophages 1–3, monocytes 1–3, dendritic cells and proliferating myeloid cells (Fig. 2a).

Monocyte 1 and macrophage 1 clusters were defined by proinflammatory features (for example, the former expressed *Ccr2*, *Cxcl2*, *Fcgr4* and *Cxcr4* and for macrophage 1 we obtained *Il1b*, *Tnfaip3* and MHC class II complex genes including *H2ab1*, *H2-Eb1*, *H2-Aa* and *Cd74*). Conversely, cluster macrophage 2 expressed regulatory macrophage markers including *Il4l1*, *Cd200r1* and *Lgals1* (Supplementary Table 1). Of note, although the proportion of both macrophage 1 and monocyte 3 clusters changed in aged tissue (Extended Data Fig. 3a) they showed no clear transcriptome variations (Supplementary Table 2). By contrast, monocyte 1 and 2 clusters showed more pronounced gene expression changes with aging, with a trend towards expression of higher levels of proinflammatory cytokine secretion and responses to proinflammatory stimuli (Extended Data Fig. 3c and Supplementary Tables 2 and 3). This included upregulation of the gene encoding IL-1β in monocyte 1 (Extended Data Fig. 3d), which is necessary for these cells to initiate a full immune response^39^. The expression of *Il1b* was also upregulated in clusters macrophage 2 and dendritic cells during aging (Extended Data Fig. 3d and Supplementary Table 2), indicating an increase in proinflammatory gene expression during aging.

Together, this indicates that myeloid cell activation exacerbates a chronic inflammatory state in aged skin.

### Increased IL-17 in the lymphoid compartment of aged skin

**Fig. 3:**
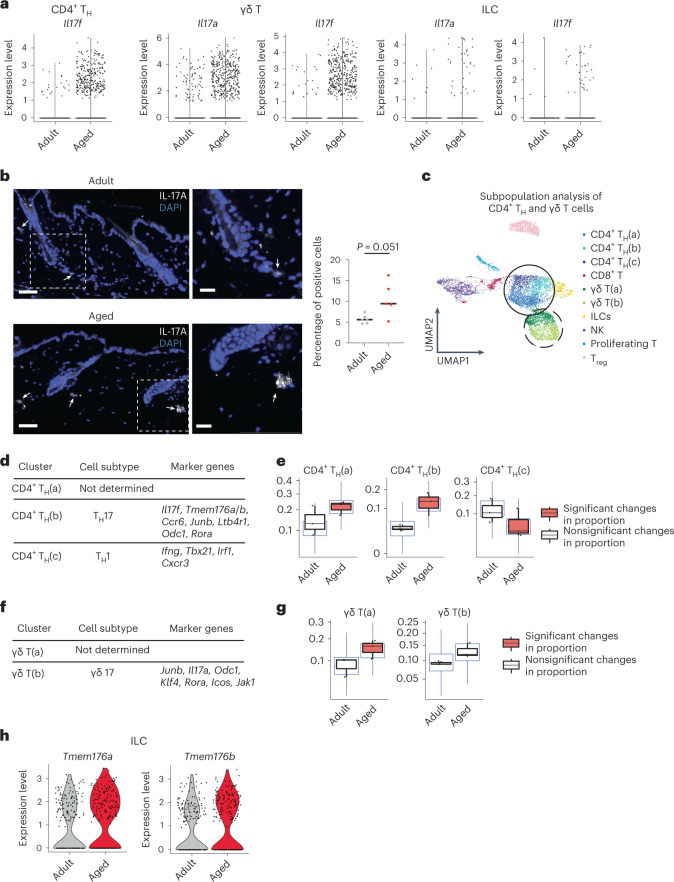
Lymphoid cells skew towards an IL-17-expressing phenotype in aging. **a**, Violin plots comparing the expression values of *Il17a* and *Il17f* in aged and adult CD4^+^ T_H_ cells, γδ T cells and ILCs. **b**, Immunofluorescence staining of IL-17A in adult (upper left) and aged (lower left) mouse back skin. Scale bars, 50 μm. Insets, higher-magnification images focusing on the dermis (right). Scale bars, 20 μm. White arrows indicate positive cells. For enhanced visualization, brightness and contrast were adjusted on these images. *n* = 6 adult mice and *n* = 5 aged mice collected in two independent experiments. Quantification is presented as the percentage of dermal positive cells. **c**, UMAP representation of subclustering of previously described CD4^+^ T_H_ cell and γδ T cell clusters. Three new clusters of CD4^+^ T_H_ cells were found (CD4^+^ T_H_(a–c)), shown here in blue and circled by the continuous line; and two new clusters of γδ T cells were found (γδ T(a) and T(b)), in green and circled by the dashed line. **d**, List of relevant markers discriminating subclusters belonging to specific CD4^+^ T_H_ cell subtypes. **e**, Boxplots depicting changes in the proportion of CD4^+^ T_H_ cell subclusters between aged and adult dermis, performed with sccomp^81^ and obtained using scRNA-seq data. Error bars indicate 95% credible intervals and center line is the median. Black boxplots represent the observed data while blue ones indicate the posterior predictive check of the model. Boxes colored orange indicate statistically significant differences in cell proportion between conditions (FDR < 0.025 using Benjamini–Hochberg procedure to control for multiple testing). **f**, List of relevant markers discriminating subclusters belonging to specific γδ T cell subtypes. **g**, Boxplots depicting changes in the proportion and γδ T cell subclusters between aged and adult dermis, performed with sccomp^81^ and obtained using scRNA-seq data. Error bars indicate 95% credible intervals and center line is the median. Black boxplots represent the observed data while blue ones indicate the posterior predictive check of the model. Boxes colored orange indicate statistically significant differences in cell proportion between conditions and red triangles mark outliers. **h**, Violin plots showing expression values of *Tmem176a* and *Tmem176b* in comparison of adult and aged ILCs. **e**,**g**, Where differences in cell proportion between ages were significant, the boxplot is marked in orange.

Lymphoid cells in the mouse dermis include T cells (CD4^+^, CD8^+^, γδ T cells and regulatory T (T_reg_) cells), ILCs and natural killer (NK) cells (Fig. 3a, left). These cells scan for tissue damage and perform immune surveillance in steady state, and also trigger an inflammatory response during wound healing and tumorigenesis^38^. The proportion of γδ T cells and CD4^+^ T_H_ cells increased in aged dermis (Fig. 2a and Extended Data Fig. 3b). We also confirmed that there was a higher frequency of CD4^+^ cells by immunostaining the dermis of aged mice (Extended Data Fig. 3e). Accordingly, γδ T cells, CD4^+^ T_H_ cells and ILCs presented the most pronounced transcriptomic changes with age from all cell types analyzed (Fig. 2b,c).

Focusing on these lymphoid populations, aged CD4^+^ T_H_ cells, γδ T cells and ILCs expressed much higher levels of inflammatory genes than their adult counterparts (Fig. 2d–f and Supplementary Tables 2 and 3). The proinflammatory cytokine interleukin 17 (IL-17) family members *Il17a* and *Il17f* were among the most strongly upregulated genes in these cell types (Fig. 2g). There are six IL-17 members, IL-17A–IL-17F, all of which signal via binding to the IL-17R family of receptors. The highly homologous cytokines IL-17A and IL-17F bind as homo- or heterodimers to IL-17RA and IL-17RC receptors^40^. The IL-17 family of cytokines intervene in tissue repair and host defense and also have pathogenic roles in autoimmune and chronic inflammatory diseases^40^. Importantly, therapeutic inhibition of aberrant IL-17A and IL-17F activities is used as treatment for skin diseases such as psoriasis and other autoimmune conditions^41–44^. Notably, during aging there is a polarization of circulating CD4^+^ T_H_ cells towards an IL-17-expressing phenotype and, in mice, γδ T cells also show this skew in peripheral lymph nodes^45–47^. However, whether these changes affect peripheral tissue aging is unknown.

**Extended Data Fig. 4:**
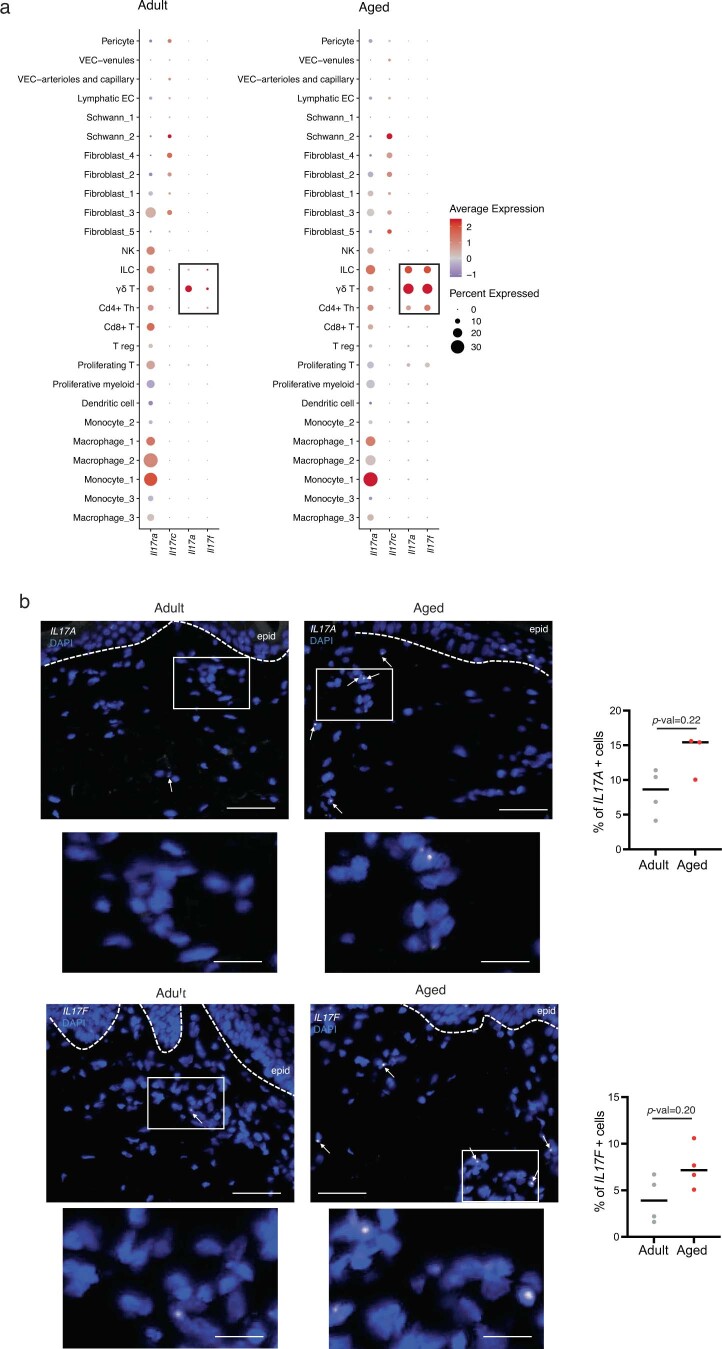
IL-17 levels in adult and aged mouse and human skin. **a**, Dot plot showing percentage of cells expressing and average expression of IL-17 receptors *Il17ra* and *Il17rc*, and ligands *Il17a* and *Il17f* in all dermal cell types. Black boxes enclose lymphoid subtypes that show differential expression of *Il17a* and *Il17f* upon aging. **b**, Fluorescent *in situ* hybridization (FISH) for *IL17A* (n = 4 individual donors for adult and n = 3 individual donors for aged) and *IL17F* (n = 4 individual donors for each age) in adult and aged human skin (top panel). Bar = 50μm. White arrows point to positive cells. Insets with higher magnification focusing on the dermis in the bottom panel, bar = 10μm. Plot shows quantification in percentage of positive cells. Brightness and contrast were adjusted only for visualization purposes. Quantifications were done in raw images.

The increase in expression of *Il17a* and *Il17f* was restricted to these specific aged lymphoid cell types (Extended Data Fig. 4a). By contrast, expression of the receptors that bind these cytokines specifically (*Il17ra* and *Il17rc*) remained mostly unaltered across all cell types during aging (Extended Data Fig. 4a). The upregulation of *Il17a* and *Il17f* in CD4^+^ T_H_ cells, γδ T cells and ILCs during aging was due to increased expression in individual cells (Fig. 3a and Supplementary Table 2), suggesting that there was a polarization towards an IL-17-expressing phenotype. We also detected, by immunostaining, a higher proportion of IL-17A-positive cells in aged mouse back skin dermis (Fig. 3b). Furthermore, we analyzed *IL17A* and *IL17F* expression in adult and aged human dermis by fluorescent in situ hybridization (FISH) to determine whether this upregulation prevailed in other species. We observed a clear trend towards a higher (although not statistically significant) percentage of *IL17A-* and *IL17F-*positive cells in aged dermis compared with adult dermis. This confirms that our results in mouse skin are, to some extent, also observed in human skin (Extended Data Fig. 4b).

In our 10X scRNA-seq data, zooming in on the CD4^+^ T_H_ cell cluster further revealed three subclusters with specific marker gene expression (Fig. 3c,d and Supplementary Table 1). While the CD4^+^ T_H_(a) cell cluster could not be assigned to any specific T_H_ cell subtype, the CD4^+^ T_H_(c) cell cluster showed markers usually expressed by T_H_1 cells such as *Ifng*. The CD4^+^ T_H_(b) cell cluster showed markers compatible with their being bona fide T_H_17 cells, including *Il17f*, *Rora*, *Tmem176a*/*b*, *Ccr6* and *Junb* among others^48–50^ (Fig. 3d). This cell subtype, CD4^+^ T_H_(b) cells (T_H_17 cells), showed an increase in abundance in aged dermis compared with control adult skin (Fig. 3e). Accordingly, the presence of CD4^+^ T_H_(c) cells (T_H_1 cells) was decreased in aged dermis (Fig. 3e), pointing again to a skewing of dermal CD4^+^ cells towards a T_H_17 cell phenotype during aging.

Similar to T_H_ cells, γδ T cells could also be further clustered into two subpopulations (Fig. 3c,f and Supplementary Table 1). The γδ T(b) cell cluster expressed markers compatible with γδ T17 cells, as indicated by the expression of *Il17a*, *Rora*, *Junb* and *Jak1* among others^50^, whereas the γδ T(a) cell cluster could not be assigned to any specific γδ T cell subtype (Fig. 3f). Both γδ T cell clusters were more abundant in aged skin although only the γδ T(a) cell subcluster was significantly increased, showing an increased presence of IL-17-expressing γδ T cells in aged mouse skin (Fig. 3g).

ILCs are crucial for the development of psoriatic pathogenesis through sustained increased secretion of IL-17 (ref. ^51^). Our analysis also showed a marked increase in the expression of genes associated with the switch to ILC subtype 3 (refs. ^51,52^), such as *Il17a*, *Il17f*, *Tmem176a* and *Tmem176b* in aged skin (Fig. 3a,h and Supplementary Table 2).

### Inhibition of IL-17A/F prevents age-related inflammation

**Fig. 4:**
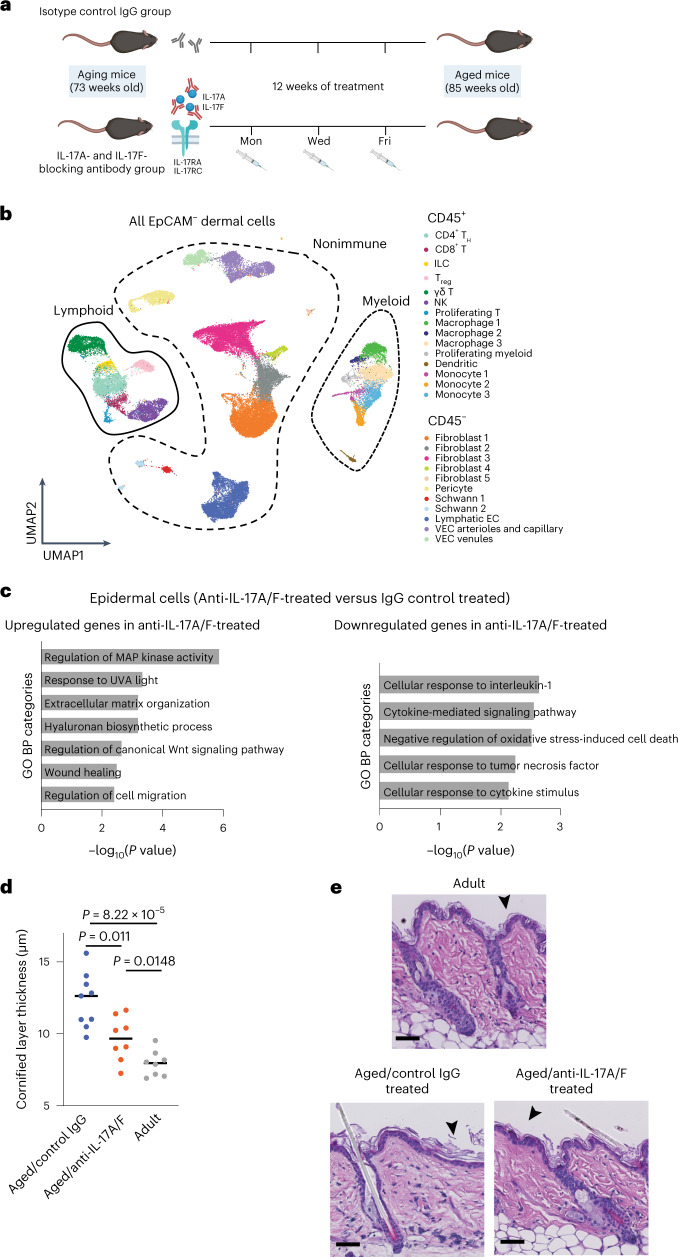
Blockade of IL-17A/F function in aging mice leads to a delay in age-associated skin traits. **a**, Diagram showing anti-IL-17A/F treatment workflow. Created with BioRender.com. **b**, UMAP representation of all CD45^+^ and CD45^–^EpCAM^–^ cells analyzed by 10X scRNA-seq obtained following IL-17A/F blockade. **c**, Plot of selected GO categories belonging to BP analysis for genes upregulated (left) and downregulated (right) after IL-17A/F-blocking treatment in epidermal cells. **d**, Dot plot showing quantification of cornified layer thickness in aged/IgG control, aged/anti-IL-17A/F-treated and adult mice. Each dot represents the average of ten measurements per mouse (*n* = 8 mice, collected in two independent experimental replicates pooled) performed on H&E stainings of skin sections. Horizontal lines represent the median of individual values per age and condition. *P* values were calculated by two-tailed Mann–Whitney *U-*test. **e**, Representative images of H&E staining of adult, aged/IgG control and aged/anti-IL-17A/F-treated mice. Scale bars, 50 μm. Cornified layer thickness (black arrowheads) was measured using scaled images.

**Extended Data Fig. 5:**
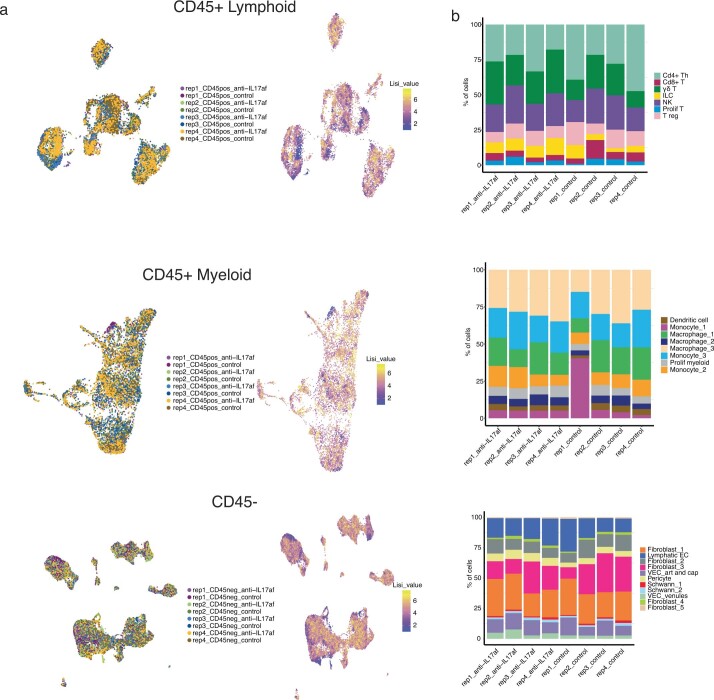
Quality controls of scRNA-seq of aged/control IG and aged/anti-IL-17 treated dermis. **a**, UMAP representation of CD45+ Lymphoid (upper panel), CD45+ Myeloid (middle panel) and CD45- (lower panel) cells showing replicate contribution and good sample mixing calculated with LISI. **b**, Bar plots showing percentage of each cell type per replicate in CD45+ Lymphoid (upper panel), CD45+ Myeloid (middle panel) and CD45- (lower panel) cells.

We next sought to determine whether increase in the expression of *Il17a* and *Il17f* contributes to the general proinflammatory state of aged skin. Treatment with antibodies that neutralize IL-17A and IL-17F (anti-IL-17A/F) is currently used as a therapy for patients with inflammatory and autoimmune diseases such a psoriasis^44^. We therefore blocked IL-17 signaling by systemic administration of anti-IL-17A/F (or an IgG isotype control) in aging mice (73 weeks old) for 12 weeks (Fig. 4a). We then isolated dermal cells by FACS and performed 10X scRNA-seq, following a previous workflow (Fig. 1a). In total, 16,975 CD45^+^ cells and 33,262 CD45^–^EpCAM^–^ cells were analyzed with the same bioinformatics pipeline as the aged sample analysis. We further verified batch effect, good sample mixing and representation of all replicates although no batch effect correction was deemed necessary for these samples (Extended Data Fig. 5a,b). We clustered the cells and confirmed that the same cell types were detected (Fig. 4b).

#### Nonimmune dermal cells

**Extended Data Fig. 6:**
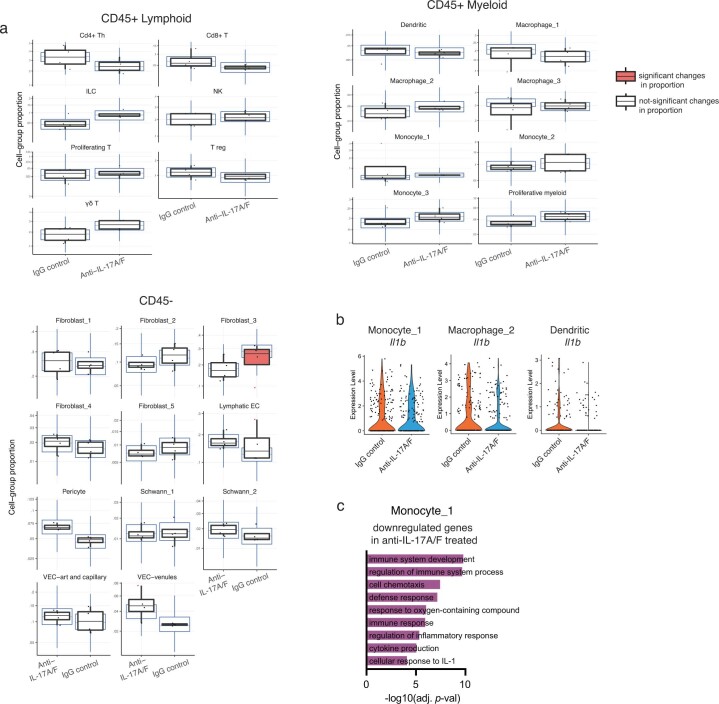
Changes observed by scRNA-seq in aged/control IgG and aged/anti-IL-17-treated dermis. **a**, Box plots showing changes in proportion of CD45+ lymphoid (upper left panel), CD45+ myeloid (upper right panel), and non-immune CD45 – (lower panel) cell populations calculated with *sccomp*(Mangiola 2022) obtained with the scRNA-seq data. Error bars indicate the 95% credible intervals, center line is the median. The black boxplots represent the observed data, while blue ones indicate the posterior predictive check of the model. Boxes colored orange indicate statistically significant differences in cell proportion between conditions (FDR < 0.025 using Benjamini-Hochberg procedure to control for multiple testing) and red triangles mark outliers. **b**, Violin plots showing expression values of *Il1b* in cells belonging to clusters monocyte_1, macrophage_2, and dendritic cells after anti-IL-17A/F treatment. **c**, Plot of selected GO categories belonging to Biological Processes (BP) analysis for genes downregulated in monocyte_1 upon IL-17A/F neutralization treatment. The x axis represents –(log_10_) of the adjusted *P*-value for each depicted GO category.

Nonimmune dermal clusters did not show major changes in gene expression following IL-17A/F inhibition. However, neutralization of IL-17A/F activity decreased the abundance of fibroblast 3 cells, previously described as the proinflammatory type of fibroblast (Extended Data Fig. 6a, bottom). This suggests that, even though the transcriptome of these cells is not markedly altered, they still responded to the blocking treatment.

#### Immune cells

Myeloid cells respond to IL-17 signaling in inflammatory situations, activating the expression of more proinflammatory cytokines^53^. The upregulation of proinflammatory cytokines (such as *Il1b* in clusters monocyte 1, macrophage 2 and dendritic cells) that we observed in aged cells was attenuated following neutralization of IL-17A/F signaling (Extended Data Fig. 6b and Supplementary Tables 4 and 5). We also observed a general decrease of proinflammatory genes in the monocyte 1 cluster (Extended Data Fig. 6c), which we previously identified as proinflammatory monocytes, although no significant changes in proportion were detected (Extended Data Fig. 6a).

Anti-IL-17A/F treatment led to no differences either at the transcriptomic level or in cell proportion analysis on lymphoid cells (Supplementary Table 4 and Extended Data Fig. 6a).

Consequently, our results indicate that blockade of IL-17A/F signaling affects certain dermal cell populations in a cell type-specific manner.

### Delay in epidermal aging traits following IL-17A/F blockade

**Extended Data Fig. 7:**
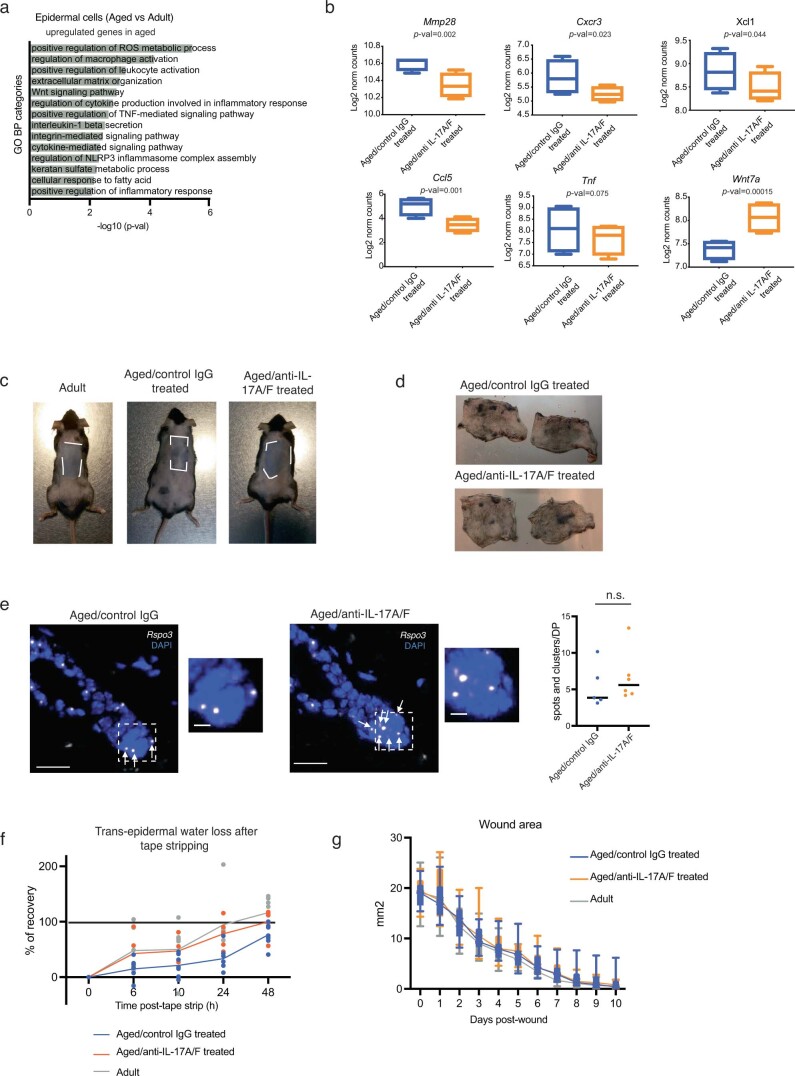
Readouts of anti-IL-17 treatment in aged mice. **a**, Plot of selected GO categories belonging to BP analysis for genes upregulated upon aging in epidermal cells. The x axis represents –(log_10_) of the *P*-value for each depicted GO category. **b**, Box and whiskers plots (min to max, box Q1 to Q3, center depicts the median) showing expression levels of selected genes differentially expressed upon IL-17A/F blocking treatment in epidermal cells (n = 4 individual mice per age). Depicted genes were extracted from the GO analysis shown in Fig. 4c. **c**, Images of adult, aged/IgG control and aged/anti-IL-17A/F antibody treated back skin showing the epilated areas (white dashed line areas). Note that the gray color of dermis indicates anagen progression. **d**, Representative images showing shaved mouse back skins with anagen patches (black areas) in aged/IgG control and aged/anti-IL-17A/F antibody treated. **e**, Representative images and quantification of *Rspo3* FISH in aged/IgG control (n = 5 individual mice) and aged/anti-IL-17A/F antibody treated (n = 6 individual mice) mouse back skin. Bar = 20μm. White arrows point to positive cells. Insets are shown in the right panel next to each condition, bar = 5μm. Plot shows the number of spots and clusters detected with *Rspo3* probe per dermal papilla. n.s., not significant. Brightness and contrast were adjusted only for visualization purposes. Quantifications were done in raw images. **f**, Plot showing percentage of recovery of trans-epidermal water loss (TEWL) after tape-stripping comparing i) adult, ii) aged/IgG control and iii) aged/anti-IL-17A/F antibody treated through time (0, 6, 10, 24, 48 hours post tape stripping). **g**, Box and whiskers plots (min to max, box Q1 to Q3) of wound area measurements (in mm^2^) through time comparing i) adult (n = 15 individual mice), ii) aged/IgG control (n = 14 individual mice) and iii) aged/anti-IL-17A/F antibody treated (n = 15 individual mice). Average of the two wounds is used for this plot.

We next investigated whether anti-IL-17A/F treatment would also ameliorate age-associated traits in keratinocytes. Sustained inflammation in aged epidermis is thought to affect epidermal stem cell fitness, potentially by reducing their regenerative function^2,10,13,17^. Bulk RNA-seq of aged and adult epidermal cells confirmed that the expression of proinflammatory cytokines and chemokine signaling increases in aged skin (Extended Data Fig. 7a and Supplementary Table 6). Importantly, IL-17A/F blockade reduced expression of cytokine-related signaling and chemotactic genes, including *Ccl5*, *Cxcr3* and *Xcl1*, as well as downregulated genes involved in proinflammatory processes including *Tnf* (Fig. 4c, Extended Data Fig. 7b and Supplementary Table 6). Concomitant to this, there was an increase in the expression of genes related to healthier wound healing such as *Wnt7a*^54^ (Fig. 4c, Extended Data Fig. 7b and Supplementary Table 6).

### Youthful skin traits increase following IL-17A/F blockade

#### Cornified layer thickness

A well-defined age-related phenotype in the epidermis is the increased thickness of the cornified layer^2^. Indeed, the thickness of this layer doubled in aged as compared with adult mice (Fig. 4d,e). In vivo systemic inhibition of IL-17 (using anti-IL-17A/F) in aging mice resulted in a thinner cornified layer compared with aged/IgG-treated control mice (Fig. 4d,e).

#### Hair follicle growth

**Fig. 5:**
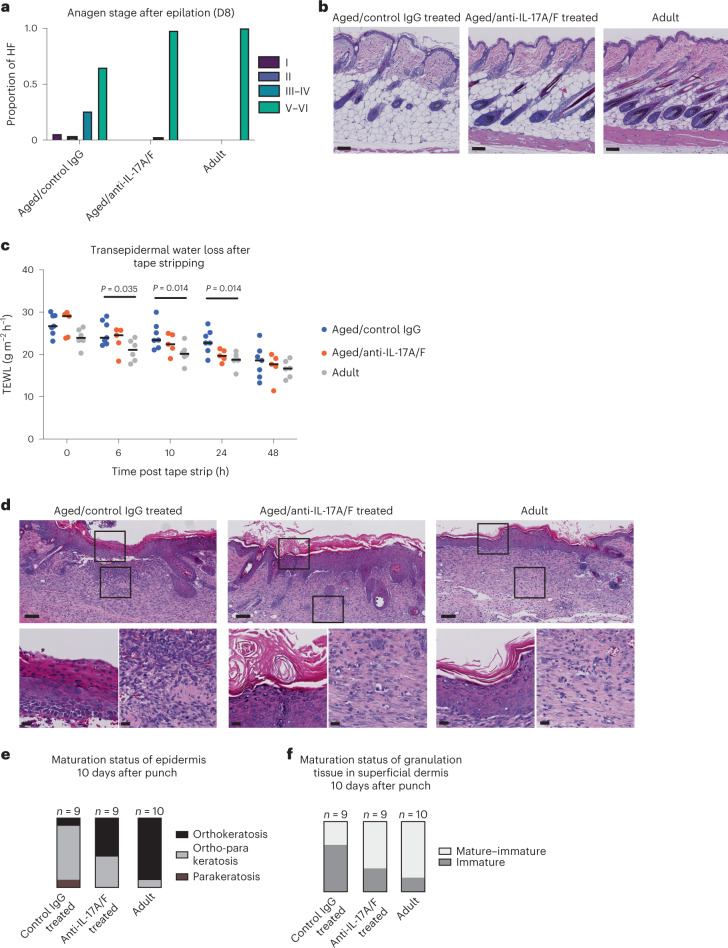
Decreased skin age-associated traits following IL-17A/F blockade. **a**, Quantification of anagen stages in mouse back skin at 8 days (D8) post epilation of aged/IgG control, aged/anti-IL-17A/F-treated and adult mice; *n* = 6 mice per condition. **b**, Details of H&E staining of epilated back skin samples at 8 days post epilation. Scale bars, 100 μm. This experiment was performed in two independent technical replicates with similar results, pooled in the analysis shown here. **c**, Plot depicting transepidermal water loss values (g m^–2^ h^–1^) after tape stripping during 48 h in aged/IgG control (*n* = 7), aged/anti-IL-17A/F-treated (n = 5) and adult (*n* = 6) mice. This experiment was performed in two independent technical replicates with similar results, pooled in the analysis shown here. *P* values were calculated by two-tailed Mann–Whitney *U-*test. **d**, Top, representative images of H&E staining of wound tissue 10 days post wound generation. Scale bars, 100 μm. Bottom, zoomed-in views of boxed areas in top panels showing details of epidermis and dermal granulation tissue. Scale bars, 20 μm. This experiment was performed in three independent technical replicates with similar results, pooled in the analysis shown here. **e**, Boxplot showing the maturation status of epidermis in aged/IgG control, aged/anti-IL-17A/F-treated and adult mice, qualified by the presence or absence of nuclei in the cornified layer (parakeratosis versus orthokeratosis) at 10 days post wounding. **f**, Boxplot showing the maturation status of granulation tissue in the superficial dermis in aged/IgG control, aged/anti-IL-17A/F-treated and adult mice at 10 days post wounding. HF, hair follicle.

Aged hair follicles show a lower capacity to enter the growing phase of the hair cycle (anagen)^17^. Anagen can be subdivided into phases that indicate how far the growth of the hair follicles has advanced^55^. As expected, hair follicles in our cohort of aged mice showed a delay in the anagen stage as compared with their adult counterparts at 8 days post epilation (Fig. 5a,b and Extended Data Fig. 7c). Although anti-IL-17A/F treatment in aged mice did not induce spontaneous anagen entry (Extended Data Fig. 7d), hair follicles of aged/anti-IL-17A/F-treated mice showed a faster pace in their anagen entry and progression almost identical to that in adult mice. This points towards a strong amelioration of the capacity of hair follicle stem cells to activate compared with aged/IgG-treated control mice (Fig. 5a,b and Extended Data Fig. 7c). Dermal papilla fibroblasts express secreted factors essential for hair follicle anagen entry, such as RSPO3 (ref. ^34^). We observed higher *Rspo3* mRNA expression by FISH in aged/anti-IL-17A/F-treated mice compared with aged/IgG-treated control mice, although this difference was not statistically significant (Extended Data Fig. 7e). This suggests that dermal papilla fibroblasts could be more fit to regrow hair following IL-17A/F blockade.

#### Transepidermal water loss

The cornified layer confers impermeability to the skin due to the presence of crosslinked proteins and lipids, and is altered during the aging process^12^. When this layer is removed by mechanical means (for example, by tape stripping), the epidermal barrier becomes permeable (higher transepidermal water loss (TEWL)). Recovery of basal TEWL values after tape stripping is achieved in a few hours under homeostatic conditions. Nonetheless, the efficiency of recovery is affected by aging^8,12,56^. Accordingly, TEWL values recorded during the first 24 h after tape stripping showed a significant delay in aged/IgG-treated mice compared with adults (Fig. 5c and Extended Data Fig. 7f), but delayed barrier recovery was not as strong in aged/anti-IL-17A/F-treated mice when compared with aged/IgG-treated mice (Fig. 5c and Extended Data Fig. 7f). This shows an improvement in the barrier recovery of aged/anti-IL-17A/F-treated mice after sublesional injury to the cornified layer compared with their aged/IgG-treated counterparts.

#### Wound healing

Efficient wound healing requires a rapid tissue response for efficient re-establishment of the skin barrier. The dialog between dermal and epidermal cells is impaired during aging, leading to a delay in wound healing^18,27^. To test the impact of in vivo IL-17A/F blockade on wound healing we created a 5-mm wound on each flank of adult, aged/IgG control and aged/anti-IL-17A/F-treated mice. Wound closure did not show differences between age groups by macroscopic measurement of wound diameter in vivo throughout the ten post-wounding days (Extended Data Fig. 7g), as suggested by previous reports^18^.

After wounding, functional barrier regeneration is achieved by complete maturation of keratinocytes, including the enucleation of the upper layers of the epidermis (orthokeratosis)^57,58^. Nevertheless, incomplete maturation of keratinocytes can occur in aberrantly healing and chronic wounds where immature nucleated cells can populate the uppermost layers of the epidermis (parakeratosis)^59^. Importantly, when we assessed the epidermal maturation status of the re-epithelized area 10 days after injury we observed that, in adult mice, most of the new epidermis was composed mainly of orthokeratotic epithelium (that is, enucleated cells in the cornified layer). Conversely, in the aged/IgG-treated control mice the presence of parakeratosis in the majority of wounds points to a delay in epidermal maturation and incorrect barrier recovery (Fig. 5d,e) previously reported for aged wounds^59^. In aged/anti-IL-17A/F-treated mice the epithelization maturation state 10 days after wounding reached a degree intermediate between adult and aged/IgG-treated control wounds (Fig. 5d,e). Moreover, in the dermis a paramount event for correct wound healing is the maturation process of granulation tissue—newly formed blood vessels, immune cells and fibroblasts that help close the wound^60^. As wound healing progresses the granulation tissue matures towards a fibrotic tissue that will eventually form a scar. We observed immature granulation tissue in the majority of aged/IgG-treated control wounds 10 days after wounding. This trend was partially reversed in adults and intermediate maturation was observed in most of these wounds (Fig. 5d,f). The blockade of IL-17A/F signaling in aged mice allowed the progression of granulation tissue maturation to an adult-like state (Fig. 5d,f).

The amelioration of these parameters during wound healing suggests that short-term in vivo blockade of IL-17A/F signaling in aging mice aids the wound-healing process at two different levels: it helps in efficient maturation of both the re-epithelized area and subjacent granulation tissue. To summarize the foregoing, aberrant IL-17A/F signaling is at the hinge between dermal and epidermal cells and its neutralization results in a synergy that delays age-associated skin traits.

### IL-17A/F–NF-κB signaling is key in epidermal aging

**Extended Data Fig. 8:**
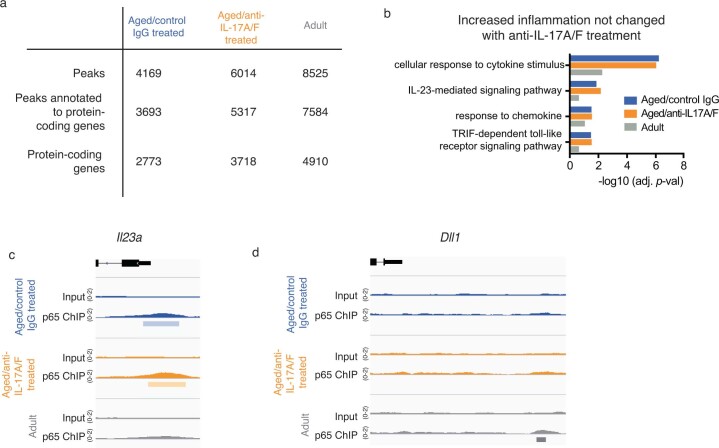
p65 ChIP in aged/control IgG and aged/anti-IL-17-treated epidermal cells. **a**, Table summarizing the number of peaks, peaks annotated to protein-coding genes and unique protein-coding genes. Results obtained in a pooled sample containing 4 biological replicates per condition (adult, aged/IgG control and aged/anti-IL-17A/F antibody treated) of the p65 ChIP-seq. **b**, Plot comparing the adjusted *P*-values of selected GO categories of genes bound by p65 in aged/IgG control, aged/anti-IL-17A/F antibody treated and adult epidermal cells. **c**, Snapshot of p65 binding to the regulatory area of *Il123a* gene in aged/IgG control, aged/anti-IL-17A/F antibody treated and adult epidermal cells. **d**, Snapshot of p65 binding upstream of the transcriptional start site of *Dll1* gene in aged/IgG control, aged/anti-IL-17A/F antibody treated and adult epidermal cells.

Epidermal cells are responders to IL-17A and IL-17F in the skin^42^. Once IL-17 receptors IL-17RA and IL-17RC become activated, they signal through downstream transcription factors such as the canonical nuclear factor κ-light-chain-enhancer of activated B cells (NF-κB)^40^. Because tight regulation of NF-κB activity is needed for correct epidermal homeostasis and defense response^38^, we hypothesized that upregulation of IL-17A/F-dependent inflammation-related genes in aged epidermis might rely on NF-κB transcriptional activity. To test this, we performed chromatin immunoprecipitation followed by sequencing (ChIP–seq) for p65 (encoded by *Rela*) in epidermal cells from aged/IgG-treated control, aged/anti-IL-17A/F-treated and adult mice. We annotated the peaks to their closest gene, filtered out those not associated with protein-coding genes and analyzed their biological function by Gene Ontology (GO) term analysis (Extended Data Fig. 8a and Supplementary Table 7).

**Fig. 6:**
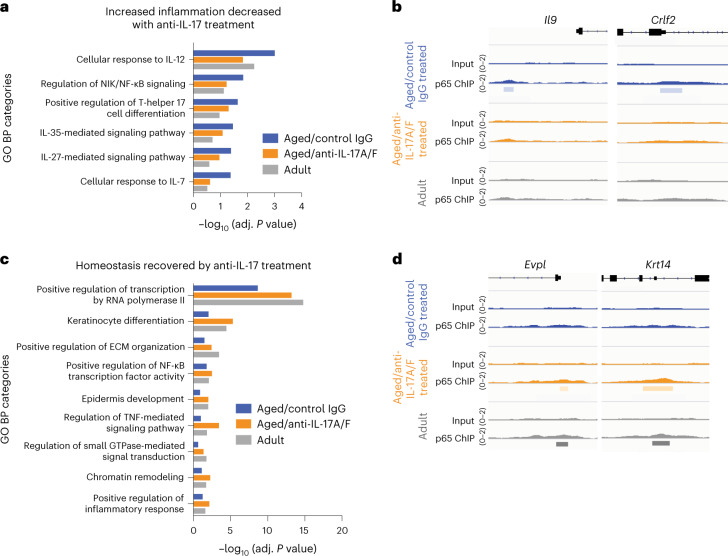
IL-17 signaling is partially transduced by p65/NFκB signaling in epidermal cells. **a**, Plot comparing the adjusted *P* values of selected inflammation-related GO categories of genes bound by p65 in aged/IgG control, aged/anti-IL-17A/F-treated and adult mice. **b**, Representative images of genes belonging to inflammation-related GO categories showing binding of p65 to their regulatory areas. **c**, Plot comparing adjusted *P* values of selected homeostasis-related GO categories of genes bound by p65 in aged/IgG control, aged/anti-IL-17A/F-treated and adult mice. **d**, Representative images of genes belonging to the selected epidermal homeostasis-related GO categories showing binding of p65 to their regulatory areas.

Genes bound by p65 in the aged/control IgG-treated samples showed an increase in inflammation-related functions when compared with those in adults, as reflected by the higher statistical significance of inflammation-related categories in GO analysis (Fig. 6a). This was accompanied by the presence of p65 in regulatory areas of specific proinflammatory genes in aged/IgG-treated control samples, such as *Il9* and *Crlf2*, which have been described as relevant in immune responses^61,62^ (Fig. 6b). After in vivo blockade of IL-17A/F activity in aging mice, this age-associated inflammatory trend was reversed (Fig. 6a,b). Nonetheless, this effect was only partial because some of these inflammatory genes remained bound by p65 following anti-IL-17A/F treatment (Extended Data Fig. 8b,c). This suggests that the effect of in vivo anti-IL-17A/F treatment in aging mice, although sufficient to delay many aging-related skin traits, may be acting only partially in a p65-binding-dependent manner.

In addition, we observed that binding of p65 to the regulatory areas of certain genes relevant to epidermal homeostasis was lost during aging (Fig. 6c). Such is the case for *Evpl* and *Krt14* (which encode envoplakin and keratin 14, respectively), essential in skin barrier function and stem cell maintenance, respectively^63,64^ (Fig. 6d). Importantly, binding of p65 to those genes was recovered following IL-17A/F in vivo blockade, suggesting that decreased IL-17A/F activity might redirect p65 to genes essential for epidermal function that are lost during aging (Fig. 6d). This effect was also partial, because other genes important for epidermal development and maturation, such as *Dll1* (ref. ^65^), remained unbound in aged/anti-IL-17A/F-treated epidermis (Extended Data Fig. 8d).

Thus, we have found that p65/NF-κB is an IL-17A/F-responsive transcription factor in epidermal cells that accounts for part of the change towards a more proinflammatory and less homeostatic gene expression in aging.

## Discussion

During aging, tissue-specific alterations in the niche synergize with stem cell-intrinsic changes to contribute to the development of age-associated traits^17,66–68^. Aging has been proposed to drive a tissue-dependent proinflammatory microenvironment that perturbs adult stem cell behavior^69–72^. Infiltration of immune cells into the stem cell niche^69^, or a transcriptional switch of stem cells, contributes to this proinflammatory environment that negatively feeds back to their own fitness^70^. Here we have characterized the effects of the proinflammatory cytokine IL-17 on skin aging. Our results show that elevated IL-17 signaling, secreted by aged dermal CD4^+^ T_H_ cells, γδ T cells and ILCs, orchestrated many of the age-associated tissue dysfunctions by exertion of pleiotropic effects. IL-17-mediated signaling is heavily linked to the development of chronic inflammatory and autoimmune diseases^42,46,73^. In the skin, these diseases include psoriasis, pemphigus and alopecia areata^43^. Even if none of the clinical signs of these diseases are common with physiological aging, they share an increased aberrant IL-17-based signaling that impedes correct skin function. Our results strongly suggest that, intriguingly, the local environment of the aged skin resembles a low-level but persistent state of chronic inflammation, with deficient permeability and impaired wound healing that is reminiscent of what occurs in serious skin diseases such as psoriasis. Consequently, anti-IL-17 therapies, already approved for treatment of psoriasis, might be repositioned to other age-associated ailments such as excessive skin dryness or difficulty in repairing damaged skin in the elderly.

## Methods

### Mouse handling and husbandry

Mice were housed under a regimen of 120h/12-h light/dark cycles and specific-pathogen-free conditions. The temperature of the animal facility was maintained between 20 and 24 °C and humidity ranged from 45 to 65%. Animals were handled following the ethical regulations and guidelines of Scientific Park of Barcelona and the Government of Catalunya. All procedures were evaluated and approved by the Ethical Committee for Animal Experimentation of the Government of Catalunya (approval reference no. 10712). All mice used were from the C57BL/6 J strain. Aged mice were either bred in-house or were retired C57BL/6 J breeder females purchased from Charles River, and were kept until the desired age in the animal facility at Barcelona Science Park. Control adult mice were either bred in-house or purchased from Charles River to generate matching cohorts. Mostly female mice were used due to this fact, and also to differences in skin-digesting efficiency between sexes, because longer male skin digestion times are required and this reduced the survival of sorted dermal cells, skewing the results towards the most resilient cell types. For experiments that did not require enzymatical digestion of the dermis, both males and females were used. Mice were always killed during darkness, to coincide with their active phase. Aged mice were between 80 and 90 weeks of age and adult mice were between 17 and 25 weeks of age. Mice with signs of skin inflammation (dermatitis, wounds or redness) were excluded from the experiments to avoid interference with the results.

### Epidermal and dermal cell isolation for genomics analysis

Mice were killed and whole-torso skin was removed as rapidly as possible. Hypodermal fat was removed by scalpel and, after two washes in PBS, skins were floated (dermal side down) in dispase II solution (5 mg ml^–1^; no. D4693, Sigma-Aldrich) in PBS for 30–40 min at 37 °C. Epidermis was removed by scalpel. For dermal cell isolation, dermis was mechanically dissociated using a McIlwain Tissue Chopper (The Mickle Laboratory Engineering Co.) and then further digested in Liberase TM (6.5 Wünsch units per reaction, Roche) diluted in DMEM (no. 41965, Thermo Fisher Scientific) for 20–30 min at 37 °C with gentle agitation. Afterwards, DNase I (1 mg ml^–1^; no. DN25, Sigma-Aldrich) was added to the mix with incubation for 15 min at 37 °C without agitation. Digested dermis was first strained through a 100-µm strainer and then through a 40-µm strainer, to obtain single-cell suspensions.

### RNA-seq

For bulk RNA-seq of adult and aged, and aged/control IgG-treated and anti-IL-17A/F-treated, mice, four mice were used per condition. Cells in single-cell suspensions were frozen in 1 ml of Trizol (Invitrogen) for posterior RNA isolation. RNA was extracted from epidermal cell pellets frozen in Trizol using the RNeasy Mini Kit (Qiagen) and further processed for mRNA-seq with Illumina sequencing technology.

### Flow cytometry and cell sorting (yielding CD45^+^, CD45^–^EpCAM^–^)

For 10X scRNA-seq, single-cell dermal suspensions were incubated with CD45–APC (clone 30-F11, 1:100, no. 559864 BD Biosciences) and EpCAM–PE (clone G8.8, 1:200, no. 552370 BD Biosciences) for 45 min on ice. After two washes in PBS, cells were resuspended in 2 µg ml^–1^ DAPI (no. 32670, Sigma-Aldrich) to stain DNA. These were then analyzed using a BD FACSAria Fusion flow cytometer with FACSDiva software v.8.0.1, in which CD45^±^ cells were retained and EpCAM^+^ cells excluded. For flow cytometry analyses, FlowJo v.10.0.8.r1 was used.

### In vivo anti-IL-17A/F-neutralizing treatment

Cohorts of aging (73-week-old) mice were randomly distributed into two groups and were treated with either (1) a mixture of 105 µg of anti-IL-17A (clone 17F3, no. BE0173, BioXCell) and 105 µg of anti-IL-17F (clone MM17F8F5.1A9, no. BE0303, BioXCell) or (2) 210 µg of control IgG1 (clone MOPC-2, no. BE0083, BioXCell). Injections of 100 µl of antibody solution in PBS were administered intraperitoneally and performed three times per week during the dark cycle at the same time of day (2–3 h into the dark phase). After 12 weeks of treatment, mice were either (1) killed to obtain samples of either dermal cells for 10X scRNA-seq or epidermal bulk RNA-seq of the epidermis and histology analysis or (2) used to measure skin aging traits.

### Epilation

Adult (*n* = 6, 18–20-week-old), aged/IgG control and aged/anti-IL-17A/F (*n* = 6, 85-week-old) female mice treated for 12 weeks with either IgG control (*n* = 6, 85-week-old) or anti-IL-17A/F were used (*n* = 6, 85 weeks old). Mice were anesthetized using a mixture of ketamine (75 mg kg^–1^ body weight) and medetomidine (1 mg kg^–1^) by intraperitoneal injection. Buprenorphine (0.05 mg kg^–1^) was injected subcutaneously as analgesic and anti-inflammatory treatment. An area of about 2–3 cm^2^ of back skin was epilated using wax papers until hair was completely removed (usually two or three rounds of waxing). Atipamezole (1 mg kg^–1^) was injected to reverse anesthesia, and mice were left on heating pads until completely recovered. Mice were housed individually to avoid scratching and contact of the epilated areas. Animal health status was monitored daily. After 8 days post hair removal, mice were killed and images of shaved back skin captured. Samples of back skin were taken, fixed in neutral buffered formalin (10%) for 3 h at room temperature, dehydrated and embedded in paraffin blocks for later histological assessment.

### Wounding and wound-healing experiments

Adult mice (*n* = 15, 18–20 weeks old) and aged mice (*n* = 14, 85 weeks old) treated for 12 weeks with IgG control as previously described, and aged male and female mice (*n* = 15, 85 weeks old) treated for 12 weeks with anti-IL-17A/F-, were used. For the IL-17-blocked group, IL-17A/F-blocking injections were stopped 1 week before starting the wound-healing assay to normalize IL-17 endogenous activity and separate the role of this cytokine in wound healing^74^ from its role during aging.

Mice were anesthetized using 3–4% isoflurane, and buprenorphine (0.05 mg kg^–1^) was injected subcutaneously for analgesia. Body temperature of the animals was maintained by placing them on a heating mat covered by a sterile surgical drape throughout the process. Hair was shaved in the back region and the area sterilized with 10% povidone-iodine in distilled H_2_O solution. Two skin biopsies were carried out per animal, one on each flank using a 5-mm-diameter sterile, disposable circular biopsy punch (5 mm diameter, Kai medical, no. BP-50F). Sharp scissors were used to specifically dissect the skin layer biopsy after using the punch, to avoid affecting other underlying tissue. Mice were placed on warm pads until fully recovered from anesthesia and then housed individually throughout the entire experiment. Wound areas were macroscopically assessed by daily measurement of length and width using a digital caliper (Traceable Digital Caliper). For wound area analysis, measurements of two wounds per animal were averaged. All wounds were collected, fixed in neutral buffered formalin (10%) for 3 h at room temperature, dehydrated and embedded in paraffin blocks for later histological assessment.

### Barrier recovery measurement

Barrier integrity was assessed by measurement of TEWL, a parameter that measures water evaporation directly on the skin surface using a probe (Tewameter TM Nano, Courage+Khazaka). The animals used included adult (*n* = 6, 18–20-week-old), aged/IgG control (*n* = 7, 85-week-old) and aged/IL-17A/F-treated (*n* = 5, 85-week-old) female mice. Back skins were shaved 24 h before barrier disruption assay. Mice were anesthetized using 3–4% isoflurane and their temperature maintained constant at 37 °C by keeping them on a tightly regulated thermal plate during TEWL measurements. The mice were left to acclimate after induction of anesthesia and before starting tape stripping and measurements. All measurements were carried out at the same location by the same person, and room temperature and humidity were recorded. The order of data collection was randomized on the first occasion but was then replicated for each consequent measure.

The epidermal barrier was disrupted by tape stripping (Corneofix CF 20, Courage+Khazaka) and the number of tape strips adjusted according to mouse age to obtain TEWL > 20 g m^–2^ h^–1^ (ref. ^75^). A range of between six and ten strips was needed for adults, and 12–16 for aged/IgG controls and aged/anti-IL-17-treated mice. For TEWL measurements the probe was used according to the manufacturer’s instructions. The probe was placed in each location of the skin surface being measured for 40 s before stabilization, each measurement lasting 1 min. TEWL probe measurements were carried out immediately after tape stripping (*t*_0_) and 6, 10, 24 and 48 h post tape stripping (*t*_6_, *t*_10_, *t*_24_, *t*_48_, respectively). Two measurements per animal were recorded, and we averaged their TEWL value to obtain one single value per animal and per time point. Also, for consistency, the probe was placed at these same two locations for each consecutive measurement. The locations used for measurements were carefully selected to avoid areas where tape stripping had caused the appearance of small wounds, to prevent alteration of the actual barrier recovery values.

### Chromatin immunoprecipitation

Four independent mice per condition of adult, aged/IgG-treated control and aged/anti-IL-17A/F-treated were used for p65 chromatin immunoprecipitation sequencing (ChIP–seq). To isolate epidermal cells, the epidermal cell isolation protocol described below was followed (Epidermal and dermal cell isolation for genomics analysis). Instead of dispase II solution, 0.8% trypsin solution (trypsin 1:250, dissolved in PBS) was used to separate dermis from epidermis for 30–40 min at 37 °C. Around 30 million pelleted epidermal cells were used per reaction. The cells were crosslinked using Gold fixative (no. C01019027, Diagenode) following the manufacturer’s directions. After two PBS washes, a second fixation was carried out in methanol-free 1% formaldehyde (no. 28908, Thermo Fisher Scientific) in MEM calcium-free medium with 10% calcium-chelated fetal bovine serum for 10 min with rotation. Glycine was then added to a final concentration of 125 mM to stop crosslinking, with incubation for 5 min under rotation. The pellet was washed twice with cold PBS and the cells then resuspended in 7.5 ml of swelling buffer (25 mM Hepes pH 7.9, 1.5 mM MgCl_2_, 10 mM KCl, 0.1% NP-40 supplemented with 1× protease inhibitors without EDTA). This cell suspension was homogenized 50 times with a Dounce homogenizer and tight pestle (no. 885302-0015, Kimble) and the extracts were centrifuged (3,000*g*, 5 min). Pelleted nuclear extracts were resuspended (in one-tenth of the volume used for the swelling buffer) in ChIP buffer (10 mM Tris-HCl pH 7.5, 150 mM NaCl, 1% Triton X-100, 5 mM EDTA, 0.5 mM DTT, supplemented with 0.2% SDS and 1× protease inhibitors without EDTA). This suspension was incubated for 15 min and then transferred to sonication tubes (no. C01020031, Diagenode). Sonication was carried out in 30 cycles of 30/30 min on/off at 4 °C (Bioruptor Pico, Diagenode) and the sonicated supernatant was centrifuged (14,000*g*, 10 min). The supernatant containing the chromatin was quantified for ChIP–seq.

For immunoprecipitation, 30 μg of chromatin was diluted with ChIP buffer without SDS to dilute the SDS concentration to 0.1% in all samples. Then, 4 μg ml^–1^ anti-p65 (no. 8242, Cell Signaling Technology) was added and samples rotated overnight at 4 °C. Sepharose beads (no. 17-5280-01, Merck) were used according to the manufacturer’s instructions, added to the tubes that included each sample and incubated with rotation for 4 h at 4 °C. Samples were then centrifuged (1,000*g*, 3 min) and washed with low (L)- and high (H)-salt buffers (L, H, L, H, L), centrifuging between washes. Low-salt buffer comprises 50 mM HEPES pH 7.5, 140 mM NaCl, 1% Triton and 1× protease inhibitors without EDTA, and high-salt buffer contains 50 mM HEPES pH 7.5, 500 mM NaCl, 1% Triton and 1× protease inhibitors without EDTA. A final wash was carried out with TE buffer (10 mM Tris-HCl pH 8.0, 1 mM EDTA). Immunoprecipitated chromatin was eluted in elution buffer (1% SDS and 100 mM NaHCO_3_, freshly prepared) by incubation of samples in the buffer for 30 min at 65 °C with agitation. Samples were then centrifuged (1,000*g*, 3 min), 5 M NaCl was added to a final concentration of 200 mM per sample followed by incubation for between 5 h and overnight at 65 °C, with agitation. Next, Tris-HCl pH 6.8 was added to a final concentration of 40 mM, EDTA to 10 mM, protease K to 50 μg ml^–1^ and incubation for 1 h at 45 °C. This product was purified with PCR columns.

#### ChIP–seq library construction and sequencing

Libraries were prepared using the NEBNext Ultra DNA Library Prep for Illumina kit (no. E7370) according to the manufacturer’s protocol. Briefly, input and ChIP-enriched DNA were subjected to end repair and the addition of ‘A’ bases to 3′ ends, ligation of the NEB adapter and USER excision. All purification steps were performed using AgenCourt AMPure XP beads (no. A63882, Beckman Coulter). Library amplification was performed by PCR using NEBNext Multiplex Oligos for Illumina (96 Unique Dual Index Primer Pairs, nos. E6440, E6442, E6444, E6446). Final libraries were analyzed using either an Agilent Bioanalyzer or Fragment analyzer High Sensitivity assay (no. 5067‐4626 or DNF‐474) to estimate the quantity and check size distribution, and were then quantified by quantitative PCR using the KAPA Library Quantification Kit (no. KK4835, KapaBiosystems). Libraries were sequenced 1 × 50 + 8 + 8 base pairs (bp) on Illumina’s NextSeq2000. Between 35 and 40 million reads were obtained per sample.

#### ChIP–seq analysis

Reads were trimmed, adapters removed and low-quality reads discarded using Trimmomatic^76^ (v.0.36, TRAILING:5 SLIDINGWINDOW:4:15 MINLEN:36). Reads were then aligned to the mm10 genome with the Burrows–Wheeler aligner^77^ (v.0.7.12, -n 2 -l 20 -k 1 -t 2). Each sample was processed individually and, once reads were aligned, the .bam files were merged and deduplicated with SAMtools^78^ (v.1.5) and downsampled to 100 million reads to obtain a pool of four replicates per condition. Peaks were called in these pooled files using MACS2 with the parameters -q 0.01–nomodel–extsize 300 -B –SPMR. Peaks were then annotated to the closest gene with Homer^79^ (v.4.11), with those peaks annotated to nonprotein-coding genes not used for further analysis.

### H&E staining

Formalin-fixed, paraffin-embedded (FFPE) blocks were cut into 2–4-µm sections and H&E staining was performed according to the standard protocol.

### Immunofluorescence

In all cases, FFPE blocks were cut into 2–4-µm sections.

IL-17A: following heat-mediated antigen retrieval (20 min at 97 °C with citrate pH 6.0), sections were blocked with 10% goat serum in PBS for 1 h at room temperature. Primary antibody incubation (anti-IL-17A, no. ab79056, abcam 1:200 in EnVision FLEX antibody diluent, Dako) was performed overnight at 4 °C. After three washes in PBS, incubation of secondary antibody (anti-rabbit Alexa Fluor 488, no. A-21206, Molecular Probes, 1:400 in EnVision FLEX antibody diluent, Dako) was carried out at room temperature for 1 h. Nuclei were counterstained with DAPI (5 µg ml^–1^ for 10 min at room temperature). Six adult and five aged mice per condition were analyzed.

CD4: antigen retrieval was performed using BOND Epitope Retrieval 2 (no. AR9640, Leica). For primary antibody incubation, rat IgG2b kappa anti-CD4 (clone 4SM95, no. 14-976-682, Thermo Fisher) was used at 1:100 and incubated for 120 min using Leica BOND RX. Nonspecific unions were blocked using 5% goat normal serum mixed with 2.5% bovine serum albumin diluted in wash buffer for 60 min at room temperature. For secondary antibody incubation, goat anti-rat IgG (H + L) and Alexa Fluor 647 (no. A-21247, Thermo Fisher) were used at 1:500 for 60 min. Nuclei were stained with DAPI and slides mounted with fluorescence Mounting Medium (no. S3023, Dako – Agilent). Five adult and five aged mice per condition were analyzed.

### FISH

For *IL17A* and *IL17F*, human adult (22–29 years old, *n* = 4, two women and two men) and aged (60–72 years old, *n* = 4, two women and two men) skin FFPE 5-µm tissue sections were purchased from Genoskin. These samples were approved by the French Ethics Committee and the French Ministry of Research and Higher Education (approval reference no. AC-2022-4863). They were then stained with RNAscope Probes—Hs-IL17A, Interleukin 17A probe (no. 310931, Bio-techne) and Hs-IL17F, Interleukin 17F probe (no. 313901, Bio-techne)—using RNAscope Intro Pack 2.5 HD Reagent Kit Red (no. 322350, Bio-techne). DAPI was used for staining of all nuclei.

For *Rspo3*, FFPE 3-µm tissue sections of mouse back skin from aged/anti-IL-17-treated or aged/IgG control mice were stained with RNAscope Probe Mm-Rspo3-O1 (no. 429861, Bio-techne) using RNAscope Intro Pack 2.5 HD Reagent Kit Red (no. 322350, Bio-techne). DAPI was used to stain all nuclei.

### Histopathological analysis

Full images were acquired with a NanoZoomer-2.0 HT C9600 digital scanner (Hamamatsu) with the ×20 objective, in which one pixel corresponds to 0.46 µm, and coupled to a mercury lamp unit L11600-05 and using NDP.scan 3.4 software U10074-03 (Hamamatsu).

Scaled images were analyzed with Qupath (v.0.3.0 and v.0.3.2). For cornified layer thickness we performed ten measurements per mouse, which were then averaged. Eight mice per condition and age were used. We always ensured that the area analyzed was in the resting hair follicle stage (telogen), to avoid interference of this parameter with the desired quantification. For wound-healing assay, qualitative analysis of dermal granulation tissue and epidermal maturation were performed in a double-blind study by histopathologists. For anagen stage assessment we assigned each hair follicle to an anagen stage based on previous reports^55^.

For immunostaining, multiple selections of the region of interest of the skin dermis, excluding the epidermis and hair follicles, were done manually in two portions of skin. The ‘positive cell count’ algorithm was used to detect positive cells. Results are presented as the percentage of positive cells. For Il-17A, NDP view2 (v.2.9.29, Hamamatsu) was used to generate snapshots from representative areas. Brightness and contrast were adjusted to help distinguish positive cells from background, using the same parameters for both conditions. For CD4 immunofluorescence, the images displayed in the manuscript were captured with an SP5 confocal microscope (Leica). Images of whole stacks were projected on a single image and brightness and contrast were adjusted to enhance the signal of positive cells, using the same parameters for both conditions. For all immunostaining, these modified images were used exclusively for visualization and never for quantification.

For *IL17A* and *IL17F* FISH, skin upper dermis—excluding the epidermis, hair follicles and skin appendages—was selected manually. In case of tissue artifacts, these were excluded from the region of interest (for example, broken areas, folds). The positive cell count algorithm was used to detect positive labeling in the TRITC channel. Results are presented as the percentage of cells with *IL17A* or *IL17F* signal (either spots—single mRNA copy—or clusters—accumulation of several mRNA copies). For visualization purposes only, representative snapshots were obtained with NDP view2 (v.2.9.29m, Hamamatsu) and brightness and contrast were adjusted to help distinguish FISH signal from background, using the same parameters for both conditions.

For *Rspo3* FISH, dermal papillae were selected manually in well-oriented telogen hair follicles. The ‘Cell detection’ algorithm was used to detect nuclei within the selected areas, and the ‘Subcellular spot detection’ algorithm to detect *Rspo3-*positive labeling in the TRITC channel in those nuclei. Individual nuclei were not always distinguishable due to high packing of dermal papillae cells. Therefore, FISH signal (spots and clusters) was not calculated per cell, but per dermal papillae. Five mice per condition were used. Graphical representations were performed with GraphPad Prism 9. For visualization purposes only, representative snapshots were obtained with NDP view2 (v.2.9.29, Hamamatsu) and brightness and contrast were adjusted to help distinguish FISH signal from background, using the same parameters for both conditions. All FISH quantifications were performed on the raw images and never on adjusted ones. Modified images were used exclusively for clearer visualization in figures.

### Bulk RNA-seq library construction and sequencing

For the adult versus aged comparison, libraries were prepared using the TruSeq Stranded Total RNA Library Prep Kit with the Ribo-Zero Human/Mouse/Rat Kit (no. RS-122-2201/2202, Illumina) according to the manufacturer’s protocol, using 150–300 ng of total RNA; for ribosomal RNA depletion, RNA was then fragmented for 4.5 min at 94 °C. The remaining steps were followed according to the manufacturer’s instructions. Final libraries were analyzed on an Agilent Technologies 2100 Bioanalyzer system using the Agilent DNA 1000 chip to estimate the quantity and validate size distribution; libraries were then quantified by quantitative R using KAPA Library Quantification Kit KK4835 (no. 07960204001, Roche) before amplification with Illumina’s cBot. Finally, libraries were sequenced on the Illumina HiSeq 2500 sequencing system using paired-end 50-bp-long reads.

For IL-17-neutralized versus control samples, libraries were prepared using TruSeq stranded mRNA Library Prep (no. 20020595, Illumina) according to the manufacturer’s protocol, to convert total RNA to a library of template molecules of known strand origin suitable for subsequent cluster generation and DNA sequencing. Briefly, 500–1,000 ng of total RNA was used for poly(A)-mRNA selection with poly-T oligonucleotides attached to magnetic beads, and two rounds of purification. During the second elution of poly-A RNA, RNA was fragmented under an elevated temperature and primed with random hexamers for complementary DNA synthesis. Cleaved RNA fragments were then copied into first-strand cDNA using reverse transcriptase (SuperScript II, no. 18064-014, Invitrogen) and random primers. Note that the addition of actinomycin D to the First Stand Synthesis Act D mix (FSA) improved strand specificity by prevention of spurious DNA-dependent synthesis while allowing RNA-dependent synthesis. Second-strand cDNA was then synthesized by removal of the RNA template and synthesis of a replacement strand, incorporating dUTP in place of dTTP to generate double-stranded cDNA using DNA polymerase I and RNase H. These cDNA fragments then had a single A base added to the 3′ ends of the blunt fragments, to prevent them from ligating to one another during adapter ligation. A corresponding single T-nucleotide on the 3′ end of the adapter provided a complementary overhang for ligation of the adapter to the fragments, ensuring a low rate of chimera (concatenated template) formation. Subsequent ligation of the multiple indexing adapter to the ends of dscDNA was carried out. Finally, PCR selectively enriched DNA fragments with adapter molecules on both ends, and the amount of DNA in the library was amplified. PCR was performed with a PCR Primer Cocktail that anneals to the ends of the adapters. Final libraries were analyzed using either Bioanalyzer DNA 1000 or Fragment Analyzer Standard Sensitivity (Agilent) to estimate the quantity and validate size distribution; libraries were then quantified by quantitative PCR using KAPA Library Quantification Kit KK4835 (Roche) before amplification with Illumina’s cBot. Finally, libraries were sequenced on the Illumina HiSeq 2500 sequencing system using single-end 50-bp-long reads.

### 10X scRNA-seq

Stained dermal single-cell suspensions were prepared as described above. CD45^+^ and CD45^–^EpCAM^–^ cells were FACS sorted separately in a BD Fusion cell sorter to enrich for the less abundant CD45^+^ cells following the sorting strategy shown in Supplementary Fig. 1a. Cells were collected in PBS + 0.5% bovine serum albumin at 4 °C in LoBind tubes (Eppendorf) and processed immediately with the microfluidics Chromium platform (10X Genomics). For the adult and aged experiment, three technical replicates were performed for CD45^+^ sorting. Replicate 1 included skin dermal cells of three adult and one aged mice, replicate 2 had two adult and one aged mice and replicate 3 comprised two adult and two aged mice. For CD45^–^EpCAM^–^ cell sorting two replicates were carried out, both comprising one mouse per condition. For aged/IL-17-blocked and aged/IgG control 10X scRNA-seq of dermal skin cells, both CD45^+^ and CD45^–^EpCAM^–^ conditions included four replicates in total. In both conditions, replicate 1 consisted of two aged/IL-17-blocked and two aged/IgG control mice. Replicates 2, 3 and 4 consisted in all cases of one aged/IL-17-blocked and one aged/IgG control mouse.

### Data preprocessing

Sequences were demultiplexed and aligned according to the Cell Ranger pipeline (v.6.0.0) with default parameters. Sequencing reads were mapped against the mouse GRCm38 reference genome to generate feature-barcode matrices separately for all CD45^+^ and CD45^–^ replicates.

### Quality control and technical bias corrections

Gene count matrixes were analyzed with the Seurat package (v.4.0.4) in R (v.4.0.3)^80^. Replicates were merged, analyzed and annotated separately for CD45^+^ and CD45^–^ datasets before integration. Cells were filtered with >10% of mitochondrial gene content and genes not found in at least five cells. As part of quality control, cells situated between the minimum and first quartile (according to the distribution of number of genes per cell of each compartment dataset) were removed. To avoid contamination of epithelial cells in the immune compartment, EPCAM^+^ cells found in CD45^+^ sorted cells were filtered out.

In addition, to remove technical bias arising after merging the different replicates we computed differentially expressed genes among replicates using the function FindAllMarkers, then we ensured that the top 500 differentially expressed genes between replicates were not present in highly variable genes (HVGs). Thus we removed the intersection between computed differentially expressed genes and HVG.

To evaluate integration of the different replicates we used the local inverse Simpson’s index (LISI) (https://github.com/immunogenomics/LISI)^30^. This score defines the effective number of samples in the local neighborhood of a cell. Under ideal mixing we would expect to obtain LISI scores equal to the number of different replicates in our datasets. This indicates that the neighborhoods are well represented by all samples and that the cell types/states previously identified exhibit good mixing across all replicates. In addition we assessed the performance of replicate integration by qualitative inspection of UMAPs. We checked that all cell types and states were well represented by each replicate and that our dataset did not contain any technical cluster entirely driven by replicate effect.

### Clustering

Cell-to-cell variations were normalized by expression values using a scale factor of 100,000 and log transformation. Gene expression measurements were scaled and centered. Scaled *z*-score values were then used as normalized gene measurement input for both clustering and visualization of differences in expression between cell clusters. HVGs were selected by assessment of the relationship of log(variance) and log(mean) and choosing those with the highest variance:mean ratio. Principal component analysis was used to reduce the dimensionality of the dataset, and ElbowGraph to select the number of dimensions for the clustering of significant principal components. Cluster identification was performed using the functions FindNeighbors and FindClusters, which calculate *k*-nearest neighbors and generate the shared nearest-neighbor graph to cluster cells. The algorithm applied was the Louvain method, which allows tuning of the number of clusters with a resolution parameter. To explore clusters in more detail, either the resolution parameter was increased in FindClusters function or FindSubCluster was used for specific clusters. UMAP was used as a nondimensional reduction method to visualize clustering.

In the case of the aged/control IgG-treated versus aged/anti-IL-17A/F-treated fibroblast clusters, we conducted a subclustering analysis of all populations obtained to detect potential fibroblast populations responding to the blocking treatment. With this we separated the previously named fibroblast 1 cluster obtained in the aged versus adult comparison into three subclusters (fibroblasts 1.0–1.2). Then, fibroblast 2 cluster was divided into two new clusters (fibroblasts 2.0 and 2.1) and fibroblast 3 cluster was separated into four clusters (fibroblasts 3.0–3.3); fibroblast 4 and 5 clusters remained as separate units. This separation allowed detection of subtle differences in the IL-17A/F blockade response in fibroblast subgroups.

### Cell type annotation

For annotation of cell types and states, enriched genes were first identified in each of the clusters with the function FindAllMarkers using the Wilcoxon rank-sum test to find cluster-specific markers. These cluster-specific genes were then explored to find previously reported cell population marker genes. Examples of marker genes used to annotate the cell populations include: CD4^+^ T cells: *Cd28* and *Cd4*; CD8^+^ T cells: *Cd8a* and *Cd8b1*; dermal cells: *Cd207*; fibroblast 1: *Crabp1*, *Inhba* and *Notum*; fibroblast 2: *Col1a1*, *Col1a2*, *Cd34*, *Robo1* and *Col3a1*; fibroblast 3: *Efemp1*, *Il1r2* and *Ccl11*; fibroblast 4: *Col11a1*, *Aspn* and *Coch*; fibroblast 5: *Myoc* and *Dcn*; ILCs: *Il13* and *Kit*; lymphatic endothelial cells: *Lyve1* and *Hes1*; macrophage 1: *Il1b*; macrophage 2: *Tnfsf9*; macrophage 3: *Ear2* and *Cd163*; monocyte 1: *Plac8* and *Cd14*; monocyte 2: *Ccl8* and *C1qa*; monocyte 3: *Retnla*, *C1qb* and *Ccr2*; natural killer cells: *Gzmc*, *Ccl5* and *Nkg7*; pericytes: *Acta2* and *Rgs5*; proliferating macrophages: *Mki67*; proliferating T cells: *Hmgb2*; Schwann cells: *Cryab, Plekha4* and *Scn7a*; Schwann cells 1: *Kcna1*; Schwann cells 2: *Sox10*; T regulatory cells: *Foxp3*; venous endothelial cell arterioles and capillaries: *Ptprb* and *Flt1*; venous endothelial cell venules: *Aqp1*, *Sele* and *Pecam1*; and γδ T cells: *Trdc* and *Trgc1*. For the annotation of IgG-treated versus anti-IL-17A/F-treated clusters we used the markers mentioned above.

### Differential expression analysis for each cluster

To find differentially expressed genes between adult versus aged, and IgG-treated versus anti-IL-17A/F-treated, in different annotated cell type populations, differential expression analysis was performed between conditions for each cluster with the function FindMarkers. To control for multiple comparisons we used the FDR Benjamini–Hochberg method.

### Age effect analysis

To assess age-related effects among immune cells, a deep learning approach was created based on autoencoders. During training this method can identify features relevant to the data structure and then use them to predict different cell types in a test dataset. To generate the model, adult data were divided into two smaller subsets that could be used as training and test sets. The model displayed high probability scores (*P* > 0.5) in the prediction of cell types from the test set among the different immune cell types and a low rate of unpredicted cells (unclassified rate <5%). If the rate of unclassified cell type is strictly biased for aged cells, this would reflect the effect of aging on the cell phenotype. Based on this assumption an age-deviance score was defined as 1 – *q*, where *q* is the probability of the unpredicted cell being in the true corresponding cell type class in aged cells. The proportion of unclassified cells within each cell type in aged cells, and the proportion of age-affected cells, were then measured and normalized by the model’s error rate (for example, by subtracting the cell proportion in each adult cell type that could not be predicted by the model).

### Cell composition analysis

To evaluate the significance of differences in cell type abundance between conditions we applied sccomp^81^, a tool designed for differential composition and variability analyses that relies on sum-constrained independent beta-binomial distributions. This model uses mean-variability association, which allows modeling of the compositional properties of the data while enabling the exclusion of outliers. We assessed significant differences (FDR < 0.025, using a Benjamini–Hochberg procedure to control for multiple testing) for composition and/or group-specific variability in each cell type population, comparing adult versus aged conditions and aged/anti-IL-17A/F-treated versus aged/IgG-treated control.

### Bioinformatics analyses of bulk RNA-seq data

FastQ files were aligned against the mm10 reference genome using STAR 2.5.2b^82^ with default options. Unless otherwise specified, all downstream analyses were performed using R 3.5.1. Differentially expressed genes (DEGs) between conditions were determined using DESeq2 1.22.1 (ref. ^83^), using mm10 gene counts as generated by the function featureCounts from the RSubread package v.1.32.4 (ref. ^84^), with options annot.inbuilt = ‘mm10’,allowMultiOverlap = TRUE,countMultiMappingReads = FALSE,minMQS = 1,ignoreDup = FALSE). Genes were selected as DEGs with the thresholds |lfcShrink foldChange | >1.25 and Benjamini–Hochberg-determined *P* < 0.1, using experimental batch as covariate. Gene set enrichment analysis was performed using gene set collections at the *Mus musculus* gene symbol level. Gene set collections used were GOBP, GOMF, GOCC and KEGG, obtained using the package org.Mm.eg.db (November 2014); GOSLIM, obtained from geneontology.org (November 2014); and Broad Hallmarks, obtained from the Broad Institute MSigDB website (https://www.gsea-msigdb.org/gsea/msigdb/) and mapped from human to mouse genes using homology information from Ensembl biomart archive (July 2016). Analyses were performed using regularized log transformation (rlog) applied to count data using the DESeq2 R package 1.22, with ROAST^85^ and the MaxMean statistic (http://statweb.stanford.edu/~tibs/GSA/).

### GO analysis

GO analysis for scRNA-seq was performed on lists ranked by increasing Benjamini–Hochberg-adjusted *P* values with g:Profiler^86^ (https://biit.cs.ut.ee/gprofiler/gost), using the biological processes database. A GO category was considered significant with adjusted *P* < 0.05. In the aged versus adult comparison, genes with fold change > 0.35 (log_2_) were considered. In the aged/anti-IL-17A/F-treated versus aged/IgG-treated control, genes with fold change > 0.25 (log_2_) were considered.

GO analysis for both epidermal bulk RNA-seq and p65 ChIP–seq was performed with enrichR^87^ (https://maayanlab.cloud/Enrichr/). A category was considered significant when *P* < 0.01.

### Statistics and reproducibility

No statistical methods were used to predetermine sample size. This was decided taking into the account variability between samples, particularly in aged mice, and was based on our expertise with the skin of aged mice^1,2,22^.

In general graphs show individual values and median (depicted as a line) and *P* values were obtained using the nonparametric two-tailed Mann–Whitney *U*-test with Prism v.9, unless otherwise stated in the figure legends.

Data collection and analysis were not performed blind to the conditions of the experiments, except for the histopathological wound healing analysis.

### Reporting summary

Further information on research design is available in the Nature Portfolio Reporting Summary linked to this article.

## Supplementary information

Reporting Summary

Supplementary Table 1. Marker expression of clusters from the CD45^–^/Epcam^–^ compartment, the CD45^+^ (lymphoid and myeloid lineages) compartment and the CD4^+^Th and gdT subclusters obtained from scRNA-seq of aged and adult dermal cells.

Supplementary Table 2. DEG analysis of clusters obtained from scRNA-seq comparing expression values for aged and adult skin.

Supplementary Table 3. GO analysis of differentially expressed genes comparing adult versus aged expression values of selected immune CD45^+^ cell clusters. The analysis is based on a BP database.

Supplementary Table 4. DEG analysis of clusters obtained from scRNA-seq comparing expression values for aged dermal cells treated with either anti-IL-17A/F-neutralizing antibodies or a control IgG.

Supplementary Table 5. GO analysis of differentially expressed genes comparing control treated versus IL-17A/F-blocking antibodies-treated expression values of selected immune CD45^+^ cell clusters (monocyte 1 cluster, genes downregulated after IL-17 blockade). The analysis is based on a BP database.

Supplementary Table 6. DEG analysis of epidermal RNA-seq comparing aged and adult expression values, and comparing control treated versus IL-17A/F-blocking antibody-treated expression values. GO analysis of differentially expressed genes comparing aged versus adult and control treated versus IL-17A/F-blocking antibody-treated epidermal cells. The analysis is based on a BP database.

Supplementary Table 7. List of peaks annotated to the closest gene. GO analysis of protein-coding genes bound by p65 in adult, aged control IgG-treated and aged IL-17A/F-blocking antibody-treated. The analysis is based on BP database.

## Data Availability

RNA-seq data are deposited at the NCBI GEO repository with accessions GSE190182 (https://www.ncbi.nlm.nih.gov/geo/query/acc.cgi?acc=GSE190182) and GSE190393 (https://www.ncbi.nlm.nih.gov/geo/query/acc.cgi?acc=GSE190393) for epidermal bulk RNA-seq data, and GSE193920 (https://www.ncbi.nlm.nih.gov/geo/query/acc.cgi?acc=GSE193920) for scRNA-seq data. ChIP–seq data are deposited at the NCBI GEO repository with accession GSE213732 (https://www.ncbi.nlm.nih.gov/geo/query/acc.cgi?acc=GSE213732). scRNA-seq analysis scripts are available at https://github.com/mereulab/IL17-SkinAging. The remaining data generated or analyzed during this study are included in this published article (and its Supplementary Information files). GO biological processes categories can be found at g:Profiler^86^ (https://biit.cs.ut.ee/gprofiler/gost).
