# Supplementary material for: Targeting lymphoid-derived IL-17 signaling to delay skin aging
Source: Nat Aging. 2023 Jun 8;3(6):688–704. doi: 10.1038/s43587-023-00431-z (PMC10275755; doi:10.1038/s43587-023-00431-z)
Supplement: Supplementary file 1 — Reporting Summary [file 43587_2023_431_MOESM1_ESM.pdf]

nature portfolio

Corresponding author(s): Salvador Aznar-Benitah, Guiomar Solanas, Holger Heyn

Last updated by author(s): Mar 29, 2023

Reporting Summary

Nature Portfolio wishes to improve the reproducibility of the work that we publish. This form provides structure for consistency and transparency in reporting. For further information on Nature Portfolio policies, see our Editorial Policies and the Editorial Policy Checklist.

Statistics

For all statistical analyses, confirm that the following items are present in the figure legend, table legend, main text, or Methods section.

n/a	Confirmed	
☐	☒	The exact sample size (*n*) for each experimental group/condition, given as a discrete number and unit of measurement
☐	☒	A statement on whether measurements were taken from distinct samples or whether the same sample was measured repeatedly
☐	☒	The statistical test(s) used AND whether they are one- or two-sided<br>*Only common tests should be described solely by name; describe more complex techniques in the Methods section.*
☐	☒	A description of all covariates tested
☐	☒	A description of any assumptions or corrections, such as tests of normality and adjustment for multiple comparisons
☐	☒	A full description of the statistical parameters including central tendency (e.g. means) or other basic estimates (e.g. regression coefficient) AND variation (e.g. standard deviation) or associated estimates of uncertainty (e.g. confidence intervals)
☐	☒	For null hypothesis testing, the test statistic (e.g. *F*, *t*, *r*) with confidence intervals, effect sizes, degrees of freedom and *P* value noted<br>*Give P values as exact values whenever suitable.*
☒	☐	For Bayesian analysis, information on the choice of priors and Markov chain Monte Carlo settings
☒	☐	For hierarchical and complex designs, identification of the appropriate level for tests and full reporting of outcomes
☒	☐	Estimates of effect sizes (e.g. Cohen's *d*, Pearson's *r*), indicating how they were calculated

Our web collection on statistics for biologists contains articles on many of the points above.

Software and code

Policy information about availability of computer code

Data collection	A BD FACSAria Fusion flow cytometer was used to sort the cells for 10X single cell sequencing, using FACSDiva version 8.0.1. Hematoxylin and eosin (H&E) images, were acquired using a NanoZoomer scanner (Hamamatsu) at 20x magnification, in which 1 pixel corresponds to 0.46um, and coupled to a mercury lamp unit L11600-05 and using NDP.scan 3.4 software U10074-03 (Hamamatsu, Photonics, France).
Data analysis	For flow cytometry analyses, FlowJo v10.0.8.r1 was used. Scaled images were analyzed with Qupath (v0.3.0 and v0.3.2). All graphical representations were performed with GraphPad Prism 9. For the RNA Sequencing data, STAR 2.5.2b, R 3.5.1, DESeq2 1.22.1, and R Subread package v1.32.4 were used. For the 10X single cell sequencing data, Cell Ranger pipeline v6.0.0 and the Seurat package v4.0.4 in R v4.0.3, were used. Local inverse Simpson’s Index (LISI) was used to ensure good sample integration. To check significance of differences in cell composition of samples, sccomp was used.

For manuscripts utilizing custom algorithms or software that are central to the research but not yet described in published literature, software must be made available to editors and reviewers. We strongly encourage code deposition in a community repository (e.g. GitHub). See the Nature Portfolio guidelines for submitting code & software for further information.

Data

Policy information about availability of data

All manuscripts must include a data availability statement. This statement should provide the following information, where applicable:

- Accession codes, unique identifiers, or web links for publicly available datasets
- A description of any restrictions on data availability
- For clinical datasets or third party data, please ensure that the statement adheres to our policy

The 10X single cell and RNA sequencing datasets generated during and/or analysed during the current study are available in the GEO repository, https://www.ncbi.nlm.nih.gov/geo/query/acc.cgi?acc=GSE190182; https://www.ncbi.nlm.nih.gov/geo/query/acc.cgi?acc=GSE190393; https://www.ncbi.nlm.nih.gov/geo/query/acc.cgi?acc=GSE193920; https://www.ncbi.nlm.nih.gov/geo/query/acc.cgi?acc=GSE213732. ScRNA-seq analysis scripts are in https://github.com/mereulab/IL17-SkinAging. Gene ontology biological processes software can be found in g:Profiler86 (https://biit.cs.ut.ee/gprofiler/gost). The rest of the data generated or analyzed during this study are included in this published article (and its supplementary information files).

Human research participants

Policy information about studies involving human research participants and Sex and Gender in Research.

Reporting on sex and gender	For this study, healthy skin from 2 adult men, 2 adult women, 2 aged men and 2 aged women. These samples were commercially available at Genoskin (France).
Population characteristics	Adults were aged 22 to 29 and aged individuals were from 60 to 72 years old. All skin samples were from healthy volunteer donors.
Recruitment	Samples were purchased from Genoskin (France), and were selected from their catalogue to match our sex and age choice.
Ethics oversight	French Ethics Committee (Comité de Protection de Personnes or CPP) and the French Ministry of Research and Higher Education

Note that full information on the approval of the study protocol must also be provided in the manuscript.

Field-specific reporting

Please select the one below that is the best fit for your research. If you are not sure, read the appropriate sections before making your selection.

☒ Life sciences ☐ Behavioural & social sciences ☐ Ecological, evolutionary & environmental sciences

For a reference copy of the document with all sections, see nature.com/documents/nr-reporting-summary-flat.pdf

Life sciences study design

All studies must disclose on these points even when the disclosure is negative.

Sample size	No statistical methods were used to pre-determine sample sizes. This was decided taking into the account the variability existing between samples, particularly in aged mice, and was based on previous experience of the authors with aged mice, especially in skin. Sample size is included in the figure legends for each experiment and/or in the methods section.
Data exclusions	Mice with skin wounds (fighting or dermatitis) were excluded due to possible interference with the data. For experiments that required enzymatic digestion, female mice were used due to differences in skin digesting efficiency between sexes, as longer male skin digestion times are required and this reduced the survival of sorted dermal cells, skewing the results towards the most resilient cell types. For the rest of experiments, balances numbers between males and females were used.
Replication	All biological replicates for each experiment are included in the figure legends and the methods section. The majority of experiments included at least three or more biological and technical replicates, except for some of the 10x single cell sequencing data, which was obtained in duplicates. We obtained similar results in all different replicates, and key results were further confirmed with additional experimental approaches.
Randomization	For the IL-17 blocking experiments, mice were randomized and changed into cages different from the ones in which they were housed prior to the experiment. Randomization was not conducted for the rest of the experiments carried out, as these were performed on already randomized animals in each condition. Animals used for the same experiments were obtained from different cages, in order to avoid generating bias.
Blinding	Blinding was not carried out in the majority of the experiments as the same investigators processed all the experiments and analysed the data. For the histopathological assessments and quantifications, the investigator analyzing the data was blinded as to the condition being quantified.

Reporting for specific materials, systems and methods

We require information from authors about some types of materials, experimental systems and methods used in many studies. Here, indicate whether each material, system or method listed is relevant to your study. If you are not sure if a list item applies to your research, read the appropriate section before selecting a response.

Materials & experimental systems

n/a	Involved in the study
☐	☒ Antibodies
☒	☐ Eukaryotic cell lines
☒	☐ Palaeontology and archaeology
☐	☒ Animals and other organisms
☒	☐ Clinical data
☒	☐ Dual use research of concern

Methods

n/a	Involved in the study
☐	☒ ChIP-seq
☐	☒ Flow cytometry
☒	☐ MRI-based neuroimaging

Antibodies

Antibodies used	anti-IL-17A (clone 17F3, #BE0173 BioXCell), anti-IL-17F (clone MM17F8F5.1A9, #BE0303 BioXCell), IgG1 (clone MOPC-2, #BE0083 BioXCell), CD45–APC (clone 30-F11, 1:100, #559864 BD Biosciences), EpCAM–PE (clone G8.8, 1:200, #552370 BD Biosciences), IL-17A (rabbit polyclonal, 1:200, ab79056 Abcam), CD4 (clone 4SM95, rat, 1:100, #14-9766-82 ThermoFisher), C anti-Rabbit Alexa Fluor 488 (donkey polyclonal, 1:400, #A-21206 Molecular Probes), anti-Rat Alexa Fluor 647 (goat polyclonal, 1:500, #A-21247 ThermoFisher) and p65 (4ug/ml, #8242 Cell Signalling Technology).
Validation	All antibodies were purchased after prior literature research, and all usage was carried out according to datasheet instructions or previously published protocols analyzing similar parameters. For IL-17A immunostaining, the antibody was used on IL-17 KO murine skin in order to validate its specificity. This antibody reacts with human and murine sample, as described by abcam. In the case of in vivo injections, antibody validation was carried out by comparing the data obtained to an irrelevant IgG1 control. IL-17A, IL-17F and IgG antibodies used for injections have been approved for murine usage by BioXCell. No further validation was carried out.

Animals and other research organisms

Policy information about studies involving animals; ARRIVE guidelines recommended for reporting animal research, and Sex and Gender in Research

Laboratory animals	Retired C57Bl/6J breeder females were purchased from Charles River and were aged until the desired age in the animal facility at the Barcelona Science Park (PCB). In some cases, when smaller cohorts were needed, aged mice were bred in-house to obtain sex-balanced groups. Control adult mice were either bred in-house or purchased to Charles River to generate matching cohorts. Aged mice were between 80- to 90-weeks of age, and adult mice were between 17-to 25-weeks of age. The temperature of the animal facility was kept between 20 to 24ºC, and the humidity ranged from 45% to 65%.
Wild animals	The study did not involve wild animals.
Reporting on sex	Mostly female mice were used due to the purchasing of aging retired breeders, and to differences between skin digesting efficiency between sexes, as longer male skin digestion times are required and this reduced the survival of sorted dermal cells, skewing the results towards the most resilient cell types. For the other type of samples not involving enzymatic digestion, both sexes were used.
Field-collected samples	The study did not involve field-collected samples
Ethics oversight	All procedures were evaluated and approved by the Ethical Committee for Animal Experimentation (CEEA) from the Government of Catalunya.

Note that full information on the approval of the study protocol must also be provided in the manuscript.

ChIP-seq

Data deposition

☒ Confirm that both raw and final processed data have been deposited in a public database such as GEO.

☒ Confirm that you have deposited or provided access to graph files (e.g. BED files) for the called peaks.

Data access links<br>*May remain private before publication.*	https://www.ncbi.nlm.nih.gov/geo/query/acc.cgi?acc=GSE213732
Files in database submission	For all samples:

Files in database submission	GSE213732_featureCounts_mm10.xlsx (count matrix)<br>GSE213732_RAW.tar (bed files)
Genome browser session (e.g. UCSC)	no longer applicable

Methodology

Replicates	4 replicates (1 individual mouse per replicate was used) per condition (adult, aged/IgG control treated, aged/anti-IL-17 treated)
Sequencing depth	All reads were 50bp, single-end.<br>Adult input R1 35789169<br>aged/IgGcontrol input R1 35314617<br>aged/anti-IL-17 input R1 36601359<br>Adult input R2 35341249<br>aged/IgGcontrol input R2 34109883<br>aged/anti-IL-17 input R2 37512657<br>Adult input R3 35642578<br>aged/IgGcontrol input R3 34839385<br>aged/anti-IL-17 input R3 40916830<br>Adult input R4 39819971<br>aged/IgGcontrol input R4 38430481<br>aged/anti-IL-17 input R4 38610390<br>Adult ChIP R1 37511829<br>aged/IgGcontrol ChIP R1 39082862<br>aged/anti-IL-17 ChIP R1 35144765<br>Adult ChIP R2 38773737<br>aged/IgGcontrol ChIP R2 33945273<br>aged/anti-IL-17 ChIP R2 40762556<br>Adult ChIP R3 34345069<br>aged/IgGcontrol ChIP R3 32243427<br>aged/anti-IL-17 ChIP R3 34629334<br>Adult ChIP R4 34478668<br>aged/IgGcontrol ChIP R4 36927691<br>aged/anti-IL-17 ChIP R4 33583864
Antibodies	p65; Cell Signaling Technology ref 8242
Peak calling parameters	Peaks were called in these pooled files with MACS2 with -q 0.01 --nomodel --extsize 300 -B –SPMR parameters; mm10 genome reference was used
Data quality	Reads were trimmed and low quality reads were dropped from the analysis.
Software	Trimmomatic (version 0.36), Burrows-Wheeler aligner (version 0.7.12), SAMtools (version 1.5), MACS2, Homer (version 4.11)

Flow Cytometry

Plots

Confirm that:

☒ The axis labels state the marker and fluorochrome used (e.g. CD4-FITC).

☒ The axis scales are clearly visible. Include numbers along axes only for bottom left plot of group (a 'group' is an analysis of identical markers).

☒ All plots are contour plots with outliers or pseudocolor plots.

☒ A numerical value for number of cells or percentage (with statistics) is provided.

Methodology

Sample preparation	In all cases, skins were floated (dermis-side down) in a Dispase II solution (5 mg/mL) in PBS for 30- to 40 min at 37ºC. Epidermises were removed with a scalpel. For dermal cell isolation dermises were mechanically dissociated and then further digested in Liberase TM (6.5 Wünsch units/reaction) in DMEM (41965) for 20 to 30 min at 37ºC with gentle agitation. Afterwards, DNase I (1 mg/ml) was added to the mix and incubated for 15 min at 37ºC without agitation. Digested dermises were strained first through a 100-µm strainer and then through a 40-µm strainer to obtain single-cell suspensions. For epidermal cell isolation epidermises were removed with a scalpel and mechanically dissociated. They were then strained through a 100-µm and then a 40-µm strainer to obtain single-cell suspensions<br><br>For 10X scRNA-seq, single-cell dermal suspensions were incubated with CD45–APC and EpCAM–PE for 45 min on ice. After two washes in PBS, cells were resuspended in 2 µg/ml DAPI to stain DNA.

Instrument	BD FACSAria Fusion flow cytometer
Software	Flow cytometry data was collected using BD FACSDiva software v8.0.1 and analyzed using FlowJo v10.0.8.r1.
Cell population abundance	For CD45+ cells, around 17% of the stained dermal population was analyzed, and CD45- cells, around 90% of that same population.<br>These percentages were dependent on the sample analyzed.
Gating strategy	In all cases, forward scatter (FSC) vs side scatter (SSC) plot was generated to separate cells from cellular debris. Then, doublets were excluded from single cells by plotting FSC-area vs FSC-width. To eliminate dead cells, FSC-area was plotted against DAPI (VIO F) channel and cells that were positive for DAPI were excluded.<br>For 10X scRNA-seq CD45+/– cells were obtained and EpCAM+ cells excluded.<br>In all cases, the gatings were set according to the corresponding FMO and single controls. An example of the sorted cells obtained is provided in Supplementary Figure 1.

☒ Tick this box to confirm that a figure exemplifying the gating strategy is provided in the Supplementary Information.

March 2021
